# Current and Emerging Prognostic Biomarkers in Endometrial Cancer

**DOI:** 10.3389/fonc.2022.890908

**Published:** 2022-04-22

**Authors:** Kelechi Njoku, Chloe E. Barr, Emma J. Crosbie

**Affiliations:** ^1^ Division of Cancer Sciences, University of Manchester, Manchester, United Kingdom; ^2^ Stoller Biomarker Discovery Centre, University of Manchester, Manchester, United Kingdom; ^3^ Department of Obstetrics and Gynaecology, St Mary’s Hospital, Manchester, University NHS Foundation Trust, Manchester Academic Health Science Centre, Manchester, United Kingdom

**Keywords:** endometrial cancer, prognosis, biomarkers, risk stratification, treatment

## Abstract

Endometrial cancer is the most common gynaecological malignancy in high income countries and its incidence is rising. Whilst most women with endometrial cancer are diagnosed with highly curable disease and have good outcomes, a significant minority present with adverse clinico-pathological characteristics that herald a poor prognosis. Prognostic biomarkers that reliably select those at greatest risk of disease recurrence and death can guide management strategies to ensure that patients receive appropriate evidence-based and personalised care. The Cancer Genome Atlas substantially advanced our understanding of the molecular diversity of endometrial cancer and informed the development of simplified, pragmatic and cost-effective classifiers with prognostic implications and potential for clinical translation. Several blood-based biomarkers including proteins, metabolites, circulating tumour cells, circulating tumour DNA and inflammatory parameters have also shown promise for endometrial cancer risk assessment. This review provides an update on the established and emerging prognostic biomarkers in endometrial cancer.

## Introduction

Endometrial cancer is the sixth most frequently diagnosed cancer in females and the gynaecological malignancy with the greatest incidence in high-income countries. In 2020, there were an estimated 417,000 incident cases and 97,000 deaths from the disease worldwide (1). The incidence of endometrial cancer is rising alongside the growing obesity epidemic (2). In the United Kingdom (UK), there are around 9,700 cases and 2,400 endometrial cancer-associated deaths every year (3). Over the last decade, deaths have increased by 25%, a trend that has been reported in other high income countries. It is projected that mortality rates for endometrial cancer will rise by a further 19% in the UK between 2014 and 2035, despite improvements in overall survival (3).

Most endometrial cancers are sporadic, with an estimated 5% occurring in the context of a hereditary predisposition, most commonly Lynch syndrome (4). Lynch syndrome is an autosomal dominant condition that arises from a defect in the DNA mismatch repair (MMR) system, predisposing to a constellation of malignancies, including endometrial cancer (5). There are currently no evidence–based screening options for endometrial cancer in either the general population or in high-risk women (6). Most women are diagnosed following routine investigations for post-menopausal bleeding, the cardinal symptom of the disease. In current clinical practice, symptomatic women are investigated by sequential tests that include transvaginal ultrasound scan, endometrial biopsy and hysteroscopy (7). Most women with endometrial cancer are diagnosed at an early stage and have highly curable disease, reflected in excellent 5-year survival rates (3). A significant minority present with adverse clinico-pathological characteristics including biologically aggressive endometrial cancer phenotypes, and have a poor prognosis. The management of endometrial cancer is primarily surgery, with total hysterectomy and bilateral salpingo-oophorectomy as standard of care worldwide. Women with high-risk features are offered adjuvant therapy with chemotherapy and/or radiotherapy, aimed at reducing risk of recurrence (8). A significant minority are managed conservatively including those of reproductive age or those for whom surgery carries considerable risk such as the frail or medically unfit (7).

Identifying those with endometrial cancer at highest risk of recurrence and cancer-related death is important to ensure women receive appropriate evidence-based care whilst avoiding the harms and costs of unnecessary treatments for those at lowest risk. Clinical, sociodemographic, histopathological and molecular factors all impact on endometrial cancer outcomes (9). A validated risk-stratification model that accurately defines risk of disease recurrence and death will guide clinical care by allowing for treatment de-escalation for those at lowest risk and intensification for those at high risk (10). Such a model may also help define the optimal follow-up programme for recurrence and guide decisions regarding alternative primary treatments for the fraction of women who are managed conservatively. This review provides an update of the current and emerging prognostic biomarkers and risk-stratification algorithms in endometrial cancer. Further, we highlight the challenges in clinical translation and offer fresh perspectives on endometrial cancer biomarker research.

## Current Endometrial Cancer Prognostic Biomarkers

### What Are Prognostic Biomarkers?

Prognostic biomarkers are clinical or biological characteristics that can be objectively assessed and evaluated to predict the course of a disease regardless of therapy (11). Prognostic biomarkers are used in clinical practice to identify the likelihood of a clinical event (mortality, disease recurrence or progression) occurring amongst those with the condition of interest (12, 13). Examples of prognostic biomarkers include clinical, tumour specific molecular and histopathological characteristics.

### Bokhman Dualistic Model of Endometrial Cancer

In 1983, Bokhman proposed a dualistic model of endometrial cancer based on clinical, epidemiologic and prognostic features (14). Type I tumours are by far the most common and are low-grade, oestrogen driven tumours that are associated with obesity and have a favourable prognosis. By contrast, type II tumours are relatively rare, high-grade, biologically aggressive tumours that are more common in healthy weight women and act independently of oestrogen (14). This model was of value several decades ago but has been shown to lack sufficient discriminatory ability to justify its continued use in the classification and management of endometrial cancers today (15). For example, ~20% of women with type I endometrial cancer experience a relapse while ~50% of those with type II do not, suggesting that the precision with which this dualistic model guides receipt of adjuvant therapy is moderate at best (16).

### Histopathological Biomarkers and Current Risk Stratification Algorithms

**Figure 1:**
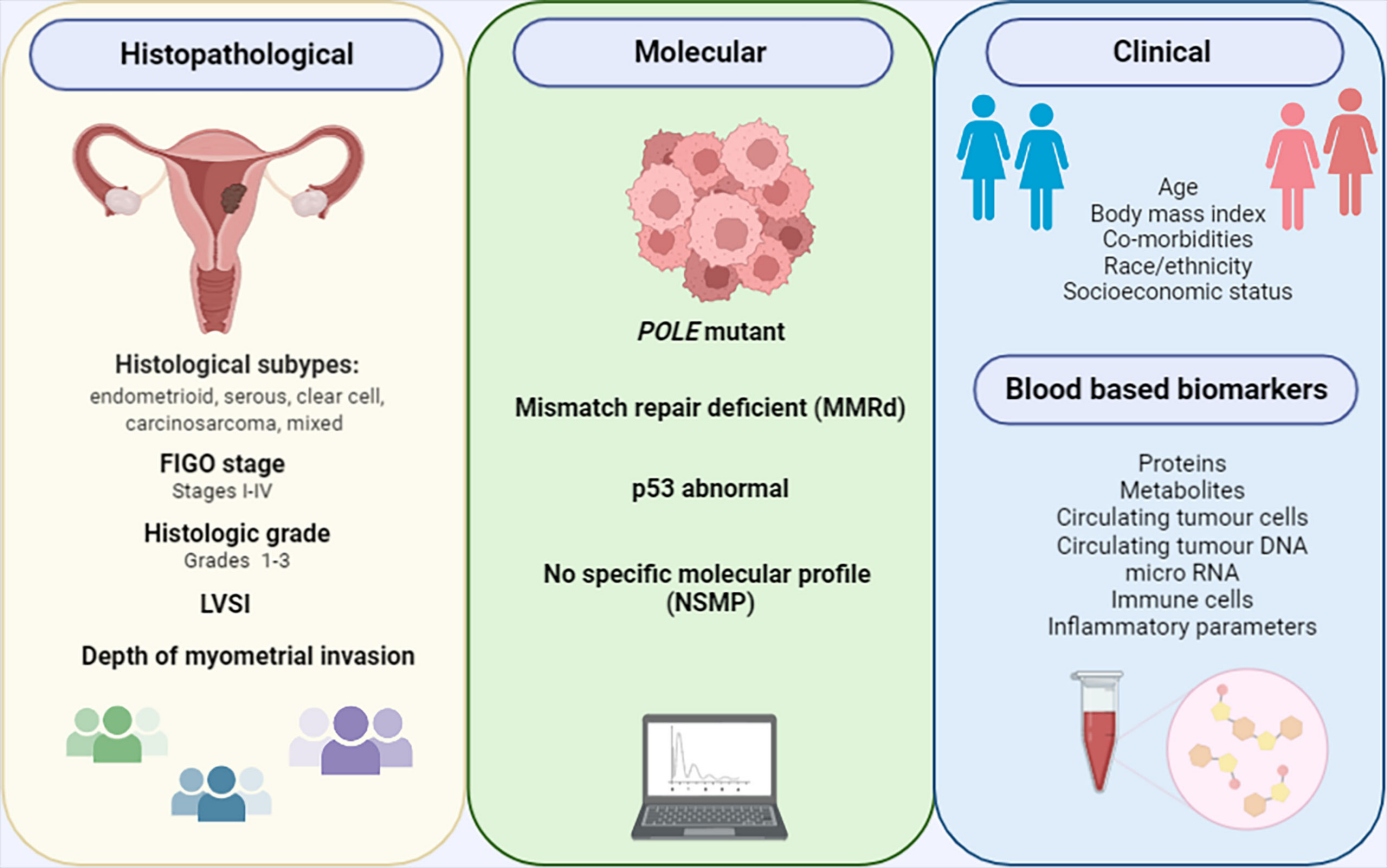
Current and emerging endometrial cancer prognostic biomarkers.

Histological subtype, FIGO stage, disease grade, presence of lympho-vascular space invasion (LVSI) and deep myometrial invasion are established prognostic biomarkers in endometrial cancer (17) (**Figure 1**). The histological subtypes of endometrial cancer include endometrioid tumours, which have a favourable prognosis, and non-endometrioid tumours (serous, clear cell, carcinosarcomas and mixed), which are biologically aggressive and associated with poor outcomes. Endometrioid tumours make up over 80% of newly diagnosed endometrial cancers, while serous, clear cell and carcinosarcomas make up 10%, 3% and <2% respectively (18, 19). Low grade endometrioid tumours are type I and high grade endometrioid and non-endometrioid histological subtypes are type II tumours. The mutational profiles of the different histological subtypes vary. *PTEN* mutations portend a favourable prognosis are more common in endometrioid endometrial cancers, while *TP53* mutations are associated with a poor prognosis and are common in serous tumours (20). Surgical staging provides important prognostic information in the management of endometrial cancer and is based on the 2009 International Federation of Gynecology and Obstetrics (FIGO) staging system (21) (**Table 1**). Women with early stage (FIGO I/II) endometrial cancer have a favourable prognosis compared to those with advanced disease (FIGO III/IV). The 5-year survival rate is >90% in early stage disease and <20% in late stage disease (17, 21). Disease grade is also an important prognostic parameter (22). Studies have been consistent in suggesting a correlation between tumour grade and depth of myometrial invasion, presence of extra-uterine disease and lymph node metastasis (23). Depth of myometrial invasion is a component of FIGO staging for stage I tumours and is an independent predictor of endometrial cancer outcomes across all stages. A recent meta-analysis of 79 studies involving 68,870 women concluded that deep myometrial invasion is associated with high endometrial cancer recurrence risk and poor outcomes (24). LVSI is also an important prognostic parameter, being linked to an increased risk of nodal spread, disease recurrence and poor outcomes (25, 26).

**Table 1:** FIGO staging of endometrial cancer (21).

FIGO Staging	Carcinoma of the endometrium
Stage I	Tumour confined to the uterus
IA	No or <50% myometrial invasion
1B	≥50% myometrial invasion
Stage II	Cervical stromal invasion, but not beyond the uterus
Stage III	Local and/or regional tumour spread
IIIA	Tumour invades serosa and/or adnexa
IIIB	Vaginal and/or parametrial involvement
IIIC	Metastases to pelvic and/or para-aortic lymph nodes
IIIC1	Pelvic node involvement
IIIC2	Para-aortic lymph node involvement ± positive pelvic lymph nodes
Stage IV	Tumour invades bladder and/or bowel, and/or distant metastases
IVA	Tumour invasion of bladder and/bowel mucosa
IVB	Distant metastases including abdominal metastases and/inguinal nodes

Adapted based on the 2009 revised staging by the FIGO Committee on Gynecologic Oncology.

Current endometrial cancer risk stratification is based on a consensus algorithm by the three major endometrial cancer consortiums: European Society for Medical Oncology, European Society of Gynaecological Oncology, and European Society for radiotherapy & Oncology (ESMO, ESTRO and ESGO) (8). This was recently updated by ESGO, ESTRO and the European Society of Pathology (ESP) to also include prognostic risk groups where endometrial cancer molecular classification information(described in detail in section 3.0) is known (27). Women are classed as low, intermediate, high-intermediate, high -risk and advanced metastatic based on histological subtype, FIGO stage, and grade, depth of myometrial invasion, presence of LVSI and molecular grouping (27) (**Table 2**). The classification system based on histopathological parameters is used to guide receipt of adjuvant treatment but has been shown to have sub-optimal ability in defining endometrial cancer outcomes (9, 28). Histological subtype and grade have poor reproducibility even amongst expert pathologists, while FIGO stage and LVSI are only available post-hysterectomy, and thus cannot inform decisions regarding surgical management (29–31). A pathology review of patients with high-risk endometrial cancer as part of the PORTEC-3 trial found significant disagreement in the assignment of several risk defining parameters including histological subtype, grade, cervical stromal invasion, LVSI and depth of myometrial invasion (32). It is therefore not surprising that the currently used risk-stratification algorithm leads to imprecise estimation of the risk of recurrence and death in women with endometrial cancer (33). Furthermore, a small minority of women with endometrial cancer are managed conservatively for fertility-sparing and surgical fitness reasons, and so cannot be surgically staged. Imaging with MRI +/-CT are limited in their ability to define risk stratifiers. Novel prognostic biomarkers that guide decisions regarding the type and suitability of alternative primary treatments in this group of women has the potential to transform patient care.

**Table 2:** Updated ESMO, ESTRO and ESGO endometrial cancer risk stratification algorithm (27).

Risk group	Molecular classification unknown	Molecular classification known
Low	Stage IA endometrioid + low-grade + LVSI negative or focal	Stage I–II ***POLE***-mutant endometrial carcinoma, no residual disease Stage IA **MMRd/NSMP** endometrioid carcinoma + low-grade + LVSI negative or focal
Intermediate	Stage IB endometrioid + low-grade + LVSI negative or focal Stage IA endometrioid + high-grade + LVSI negative or focal Stage IA non-endometrioid (serous, clear cell, undifferentiated carcinoma, carcinosarcoma, mixed) without myometrial invasion	Stage IB **MMRd/NSMP** endometrioid carcinoma + low-grade + LVSI negative or focal Stage IA **MMRd/NSMP** endometrioid carcinoma + high-grade + LVSI negative or focal Stage IA **p53abn** and/or non-endometrioid (serous, clear cell, undifferentiated carcinoma, carcinosarcoma, mixed) without myometrial invasion
High-intermediate	Stage I endometrioid + substantial LVSI regardless of grade and depth of invasion Stage IB endometrioid high-grade regardless of LVSI status Stage II	Stage I **MMRd/NSMP** endometrioid carcinoma + substantial LVSI regardless of grade and depth of invasion Stage IB **MMRd/NSMP** endometrioid carcinoma high-grade regardless of LVSI status Stage II **MMRd/NSMP** endometrioid carcinoma
High	Stage III–IVA with no residual disease Stage I–IVA non-endometrioid (serous, clear cell, undifferentiated carcinoma, carcinosarcoma, mixed) with myometrial invasion, and with no residual disease	Stage III–IVA **MMRd/NSMP** endometrioid carcinoma with no residual disease Stage I–IVA **p53abn** endometrial carcinoma with myometrial invasion, with no residual disease Stage I–IVA **NSMP/MMRd** serous, undifferentiated carcinoma, carcinosarcoma with myometrial invasion, with no residual disease
Advanced metastatic	Stage III–IVA with residual disease Stage IVB	Stage III–IVA with residual disease of any molecular type Stage IVB of any molecular type

Focal LVSI refers to the presence of a single focus around the tumour. Key: p53abn, p53-abnormal; MMRd, MMR-deficient; NSMP, no specific molecular profile.

## Emerging Endometrial Cancer Prognostic Biomarkers

### TCGA Endometrial Cancer Molecular Classification

Molecular subtyping offers a more objective and reproducible classification of endometrial cancer when compared with histopathological evaluation and has the potential to revolutionise patient care (33). Recently, the TCGA proposed four distinct endometrial cancer molecular subgroups based on mutational burden, microsatellite instability and copy number alterations observed in 373 endometrial cancer cases: copy number high, copy number low, MSI hypermutated, and *POLE* ultra-mutated (34) (**Table 3**). This classification has been validated in subsequent studies and shown to have prognostic and therapeutic implications (29, 38–41).

**Table 3:** Characteristics of the TCGA molecular classification of endometrial cancer.

Type	*POLE*(ultramutated)	MSI (hypermutated)	Copy number low (endometrioid)	Copy number high (serous like)
Prevalence	7%	28%	39%	26%
Mutation frequency	Very high (>100 mutations/Mb)	High 100-10 mutations/Mb	Low <10 mutations/Mb	Low <10 mutations/Mb
Commonly mutated genes	*POLE* (100%), *PTEN* (94%)	*PTEN* (88%) *PIK3CA* (54%)	*PTEN* (77%) *CTNNB (52%)*	*TP53* (92%) *PIK3CA (47%)*
Copy number aberrations	Very low	Low	Low	High
MSI/*MLH1* methylation	Mixed high and low MSI, stable	High MSI (*MLH1*, *PMS2*, *MSH2*, and/or *MSH6* deficiency)	MSI stable	MSI stable
Histological subtype	Endometrioid	Mostly endometrioid	Endometrioid	Serous, 25% high-grade endometrioid and mixed
Grade	G1-3	G1-3	G1-2	G3
Other features	Ambiguous histo-morphology Dense immune infiltrates	Display tumour-infiltrating lymphocytes	*CTNNB* mutations are associated with poor prognosis Subgroup with amplification of chromosome arm 1q has poor prognosis	Similar to high-grade serous ovarian carcinoma L1 cell adhesion molecule (L1CAM) expression associated with poor prognosis
Prognosis	Good	moderate	moderate	Poor

Adapted from (35–37).

The copy number high (serous-like) cancers have the worst progression-free survival and are characterised by widespread genomic alterations with extensive copy number aberrations (34, 35). Patients in this subgroup have mostly high-grade and biologically aggressive tumours including serous endometrial cancers and 25% of the grade 3 endometrioid tumours (34, 35). Mutations commonly observed in copy number high tumours include those in *TP53* and *PIK3CA*. Other mutations involving *FBXW7* and *PPP2RIA* are unique to copy number high tumours (34). Amplifications of *CCNE1* and *ERBB2* are also commonly observed (42, 43).

Copy number low endometrial cancers have few copy number aberrations and no increased mutation burden.They comprise low grade, microsatellite stable, endometrioid tumours (34, 35). Whilst tumours in this subgroup generally have a favourable prognosis, they have specific unique molecular features that are associated with poor prognosis, namely *CTNNB1* mutations and amplification of chromosome arm1q, thus making the group an interesting one for future stratified clinical trials (44, 45).

Microsatellite instable endometrial cancers have mismatch repair deficiency (MMR-d), high mutation rates and few copy number aberrations (34). They are characterised by mutations or epigenetic silencing affecting the MMR genes *MLH1*, *MSH2*, *MSH6*, and *PMS2*. Other commonly mutated genes in this sub-group include *PTEN*, *ARIDIA*, *PIK3CA*, *PIK3RI*, and *RPL22* (34, 35). These tumours are usually endometrioid although their histological morphology can be unusual, making characterisation challenging (35).

The final subgroup of the TCGA classification is the *POLE* ultra-mutated group. This subgroup is characterised by high mutation rates and hotspot mutations in the *POLE* exonuclease domain (EDM) of polymerase-έ (34). *POLE* ultra-mutated tumours exhibit few copy number aberrations and have mutations in *PTEN, PIK3RI, PIK3CA, FBXW7* and *KRAS* genes. These tumours have an excellent prognosis with the best progression free survival (46). They are characterised by dense immune cell infiltrates. Whilst previously thought not to recur, there is emerging evidence that the *POLE* tumours can recur but at a much lower rate compared to other molecular subtypes (35, 46). The recent proteogenomic characterisation of endometrial cancer by the National Cancer Institute’s Clinical Proteomic Tumour Analysis Consortium (CPTAC) provides further insights into the proteomic markers of endometrial cancer clinical and genomic tumour subgroups (47).

Whilst the TCGA classification substantially advanced our understanding of the molecular diversity of endometrial cancer and the associated prognostic implications, its clinical applicability in terms of refining surgical staging, guiding decisions about adjuvant therapy and intensity of post-treatment surveillance is limited (35). Barriers include the need for fresh-frozen tumour specimens, high costs and technical and methodological complexities.

### Simplified and Pragmatic Endometrial Cancer Molecular Classifiers

Novel molecular classification tools have been developed and validated based on the use of surrogate markers to define four distinct subgroups of endometrial cancer that are analogous but not identical to the TGCA classification (40). The classifiers include the TransPORTEC (48) and ProMisE models (49).These novel classifiers utilise immunohistochemistry to identify MMR and p53 abnormalities and targeted sequencing to identify *POLE* mutations (40, 48). In contrast to the fresh-frozen tumour specimens required for TCGA classification, these pragmatic classifiers can be used on formalin-fixed, paraffin-embedded tumour materials, thus enhancing their clinical utility (29). There is good evidence to support their potential applicability to endometrial biopsy and curettage diagnostic specimens (50–52) and the inter laboratory concordance is high (51). Studies have been consistent in confirming the prognostic value and potential clinical utility of these classifiers across unselected patient populations (53–56).

**Figure 2:**
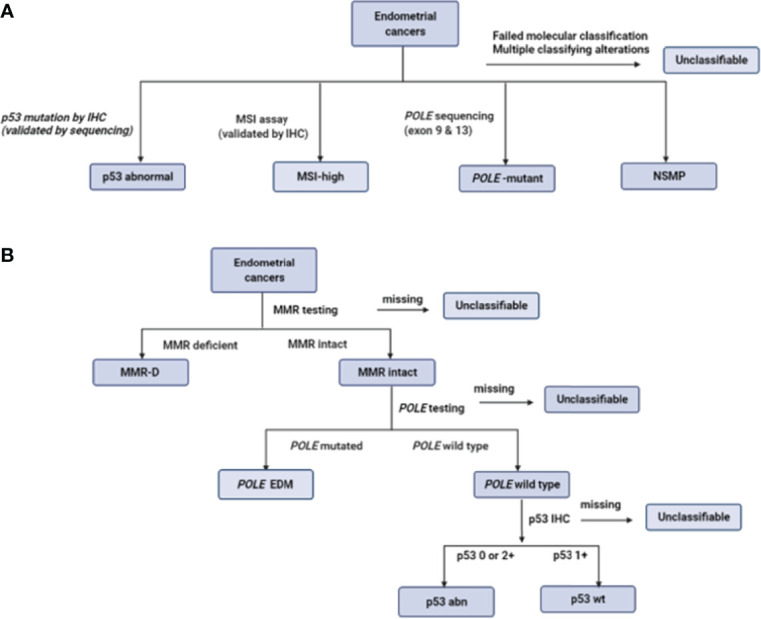
Defining the molecular subgroups of endometrial cancer based on the TransPORTEC classifier **(A)** and ProMisE **(B)**. Adapted from (48, 49, 51).

In the TransPORTEC initiative, the four molecular subgroups are p53-abnormal, MSI-high, *POLE*-mutant and those with no specific molecular profile (NSMP) (48) (**Figure 2A**). Of 116 high-risk endometrial cancer specimens analyzed by the TransPORTEC group, p53-abnormal (n=36) and NSMP (n=44) subgroups had significantly higher rates of distant metastases and lower 5-year relapse free survival than MSI-high (n=19) and *POLE*-mutant (n=14) tumours (48) (**Table 4**). The 5-year recurrence-free survival rates were 93% and 95% for the *POLE*-mutant and MSI-high subgroups respectively, compared with 42% (p53-abnormal) and 52% (NSMP) (48). A refined version of the TransPORTEC classifier has since been developed that incorporates the presence of LVSI and other molecular parameters such as L1CAM expression and the presence of *CTNNB1* mutation (57). This model is being prospectively tested in a cohort of women with high-to-intermediate risk endometrial cancer as part of the PORTEC-4a trial.

**Table 4:** Prognostic performance of ProMisE and TransPORTEC classifiers, adapted from (49) and (48), respectively.

Subgroups	N (%)	Overall survival		Disease specific survival		Progression free survival	
ProMisE		HR(95%CI)	LRT p	HR(95%CI)	LRT p	HR(95%CI)	LRT p
p53 wt	139 (45.6%)	** **Comparator group					
MMR-D	64 (20.1%)	1.90 (0.88-4.04)	0.0211	1.32 (0.51-3.35)	0.0156	0.64 (0.25-1.60)	0.011
*POLE* EDM	30 (9.4%)	1.01(0.26-2.99)		0.42 (0.04-1.88)		0.19 (0.02-0.81)	
P53 abn	86 (27.0%)	2.61 (1.27-5.72)		2.28 (1.02-5.58)		1.75 (0.84-3.96)	
**TransPORTEC**		**5-year overall survival**		**Distant recurrence rates**		**5-year recurrence free survival**	
NSMP	44 (38%)	61%	<0.001	39%	<0.001	52%	<0.001
MSI-high	19 (16%)	63%		0%		95%	
*POLE* mutant	14 (12%)	93%		0%		93%	
p53 abnormal	39 (34%)	40%		50%		42%	

ProMisE data are based on multivariable analysis in a validation cohort of 319 cancers. Variables included in model are age, BMI, grade, histology, any treatment received. TransPORTEC data included 116 high risk endometrial cancer patients. HR, hazard ratio; LRP, likelihood ratio test.

ProMisE stratifies women with endometrial cancer based on sequential molecular testing for aberrations in the order of MMR-D, *POLE* mutation and p53 status (**Figure 2B**). The four molecular groupings based on ProMisE are MMR-deficient (MMRd; analogous to MSI-high subgroup), *POLE EDM* (analogous to *POLE* ultramutated), p53-abnormal (p53 abn, analogous to the copy number high group) and p53-wild type (p53 wt, analogous to the copy number low group) (40, 49). These molecular subgroupings have also been shown to correlate with disease-free and overall survival even after adjusting for known risk parameters (35, 49). Women in the p53 abn group have the worst prognosis with a 3- to -5 fold higher risk of mortality or progressive/recurrent disease than the p53 wt group, and a 2-fold higher risk following adjustment for clinico-pathological parameters (35, 49). Those in the MMR-D subgroup have a 1.5 to 2-fold increase in mortality compared with the p53 wt subgroup; the survival benefit was non-significant following adjustment for confounding. The *POLE* EDM subgroup have the best prognosis and are least influenced by clinico-pathological features (35, 49).

### Other Molecular Prognostic Parameters and Risk Algorithms

Other molecular parameters that are prognostic in endometrial cancer include overexpression of L1CAM and loss of oestrogen (ER) and/or progesterone receptors (PR), both of which are linked to a higher risk of recurrence and death (58–61). L1CAM expression strongly correlates with non-endometrioid histology, LVSI and lymph node metastasis (58). Loss of ER/PR expression is linked to high-grade disease, deep myometrial invasion and lymph node metastasis (62). DJ-1 protein distinguishes low-grade from high-grade endometrial cancer (63) while *CTNNBI* mutations have shown potential in identifying those low-grade, early stage, endometrial cancers at higher risk of recurrence and death (44).

A number of risk-prediction models, incorporating clinical, histological and molecular parameters, have been developed to aid prediction of survival outcomes in endometrial cancer. ENDORISK, a validated risk algorithm based on four pre-operative molecular markers, namely L1CAM, PR, ER, and p53 status, predicted risk of lymph node metastasis and survival in a multi-centric cohort of 763 women with endometrial cancer across Europe, and 2 independent cohorts from the Netherlands and Norway (64). In a similar study, a model incorporating L1CAM, PR, ER and p53 status demonstrated a 48% sensitivity and 89% specificity for high-risk endometrial cancer (65). Ravegnini and colleagues found better stratification of NSMP patients with *CTNNB1* mutation alongside miR-499a-5p status (66).

### Therapeutic Implications and Additional Benefits of the Molecular Classification of Endometrial Cancer

The molecular classification of endometrial cancer has prognostic and therapeutic implications. The p53-abnormal endometrial cancers are the most biologically aggressive and would ideally be managed with complete/aggressive surgical treatment. These tumours generally require adjuvant treatment. A retrospective molecular analysis of the PORTEC-3 trial for high-risk endometrial cancer confirmed that women with p53-abnormal endometrial cancer had significantly improved recurrence-free survival when platinum-based chemotherapy was used alongside radiation, compared with radiation alone (67). This survival benefit was not observed in the other molecular categories, although the PORTEC-3 trial was not originally powered for these subgroup analyses (67). The finding of several molecular similarities between the TCGA p53 endometrial cancer group and both high grade serous tubo-ovarian cancer (HGSOC) and basal-like breast cancer, has sparked interest in the potential for therapeutics that target homologous recombination in these tumours (33, 34). A number of clinical trials assessing the efficacy of PARP inhibitors alone or in combination with anti-angiogenics/immune checkpoint inhibitors for recurrent or metastatic endometrial cancer are under way (68). The TransPORTEC Refining Adjuvant treatment IN endometrial cancer Based On molecular features (RAINBO) suite of clinical trials is evaluating the role of adjuvant chemo-radiation with or without a DNA damage response targeting agent in women with p53-abnormal endometrial cancer (39). Women with p53 wild type disease have lower metastatic potential and surgical treatment alone may suffice (69). Those with POLE mutant tumours have such a good prognosis that adjuvant treatment is unlikely to improve survival outcomes and de-escalation of therapy may be appropriate. The MMR-D molecular group is highly immunogenic, providing therapeutic opportunities for the use of immunotherapy. Marebella and colleagues, in the KEYNOTE-158 study reported an objective response rate of 57.1% in 49 endometrial cancer patients with previously treated unresectable or metastatic MMR-D disease who were treated with pembrolizumab (70). The GARNET trial, a phase 1b trial of anti-PD1 dostarlimab reported an objective response rate of 42.3% for women with recurrent or advanced MMR-D endometrial cancer that had progressed after treatment with platinum-based chemotherapy (71). Both pembrolizumab and dostarlimab have been FDA approved (71, 72).

The incorporation of endometrial cancer molecular testing into routine clinical care has several additional advantages. It will allow for the early identification of women with an inherited defect affecting one of the four MMR genes (Lynch syndrome) for whom cancer surveillance and aspirin chemoprevention may help to prevent future cancers, and cascade testing may identify other affected family members (5). For women of reproductive age who are considering non-surgical management, molecular classification of endometrial biopsy specimens can guide treatment decisions as p53 abnormal status would discourage a conservative approach to management (69).

## Blood-Based Endometrial Cancer Prognostic Biomarkers

A blood-based prognostic biomarker has strong appeal to clinicians and patients alike. ‘Can a blood test be used in predicting survivorship and/or recurrent disease?’ ranked 5^th^ most important research priority in the James Lind Alliance endometrial cancer priority setting partnership, representing the views of patients, clinicians, and members of the general public (73). A blood-based test that can accurately detect deep myometrial invasion and lymph node metastasis pre-operatively could inform surgical management. Such a test may also have utility in risk stratifying within endometrial cancer molecular groups, since women whose tumours fall within MMR-D or NSMP groupings have overlapping survival outcomes and adjuvant therapy may be beneficial for some but not all (74). Several blood-based biomarkers, including proteins, metabolites, circulating tumour cells, cell-free DNA, immune cells and inflammatory parameters have shown potential for refining endometrial cancer risk assessment. However, the evidence to enable clinical translation is limited.

The most commonly reported blood-based protein prognostic markers include cancer antigen 125 (CA125) and Human Epididymis protein 4 (HE4) (75, 76). Serum CA125 was first shown to be elevated in women with recurrent and advanced endometrial cancer by Niloff and colleagues in 1984 (77). Subsequent studies have been consistent in suggesting an association between serum CA125 concentration and adverse endometrial cancer clinico-pathological parameters and outcomes (78–82). Jiang and colleagues, in an analysis of 995 patients with endometrial cancer, found that elevated CA125 significantly correlated with lymph node metastasis, myometrial invasion, FIGO stage but not histological subtype, and was an independent prognostic factor (83). This study was limited by its retrospective design and selection bias, as almost 20% of endometrial cancer patients were excluded due to lack of pre-operative serum CA125 (83). There is good evidence of an association between serum HE4 levels and endometrial cancer outcomes. The meta-analysis by Dai and colleagues, involving 4235 patients, reported that elevated HE4 levels were significantly associated with worse overall, disease-free and progression-free survival (84). Serum HE4 has also been shown to correlate with adverse endometrial cancer histopathological parameters, although the evidence has been limited by marked heterogeneity across the various studies, small sample sizes and significant variation in the prognostic thresholds used (74). Several blood-based metabolites have also been linked to adverse endometrial cancer clinico-pathological factors and poor outcomes (13). As yet, none have been translated into routine clinical practice.

There is emerging evidence of a correlation between circulating cell-free tumour DNA levels and endometrial cancer prognosis (85–88). Cicchillitti and colleagues found elevated levels of cell-free DNA in grades 2 and 3 endometrial cancer compared to grade 1 disease (86). These findings align with the report by Vizza and colleagues of a significantly increased level of total cell-free DNA in high grade endometrial cancer (85). In addition, serum DNA integrity (the ratio between long and short cell free DNA fragments) was found to be higher in women with LVSI (85). Tanaka and colleagues, on the other hand, did not find a significant change in cell-free DNA by endometrial cancer grade or stage (89). Further studies are thus needed to confirm the potential prognostic utility of circulating tumour DNA in endometrial cancer. Circulating tumour DNA have also been suggested as potential tools for the early detection of recurrence in endometrial cancer (88, 90). The small pilot study by Moss and colleagues found that ctDNA could detect endometrial cancer recurrence and progression earlier than imaging or clinical presentation with a median lead time of 2.5 months (88). Specific blood-based tumour mutations have also been associated with endometrial cancer prognosis. Dobrzycka and colleagues found an association between circulating cell-free DNA p53 antibody and *KRAS* mutation status and high-grade endometrial cancer (87). Bolivar and colleagues found a significant association between the presence of plasma ctDNA mutation (*CTNNBI, KRAS, PTEN*, or *PIK3C*) and advanced stage, deep myometrial invasion, LVSI, and primary tumour size (91). Circulating tumour cells have also been linked to endometrial cancer prognosis. Lemech and colleagues, in a feasibility study of 30 patients with advanced endometrial cancer found an association between circulating tumour cell positivity and non-endometrioid histology, tumour size, disease stage and survival (92). The small prospective study by Bogani and colleagues, involving 28 patients with grade 3 endometrial cancer reported a significant correlation between the presence of circulating tumour cells and deep myometrial invasion and lymph node positivity (93). Studies exploring how best to incorporate circulating tumour markers into routine clinical care are needed.

Systemic inflammatory parameters have shown potential as prognostic biomarkers in endometrial cancer (94). Chronic low-grade inflammation is one of the biological mechanisms underpinning endometrial carcinogenesis. Inflammation is known to damage DNA and potentiates pro-proliferative and anti-apoptotic processes that contribute to tumour development and progression. A recent study from our group found that women with elevated CRP at a decision threshold of 5.5mg/L had a two-fold increase in cancer-specific mortality risk (95). These findings need to be validated in an independent cohort prior to clinical translation. Other inflammatory parameters that are prognostic in endometrial cancer include neutrophil to lymphocyte ratio, monocyte to lymphocyte ratio, systemic inflammatory index, and Glasgow prognostic score (**Table 5**). However, there is insufficient evidence to enable clinical translation at present.

**Table 5:** Circulating endometrial cancer prognostic biomarkers.

Category	Biomarker	Prognostic features
Proteins	Elevated CA125	Linked to poor survival (96, 97) Higher stage (83, 98) Higher grade (83, 98) Deep myometrial invasion (83, 98) Lymph node metastasis (83, 98) LVSI (98)
	Elevated HE4	Poor overall, disease-specific and recurrence free survival (74, 84) Deep myometrial invasion (99, 100) Advanced stage (100–102) Presence of LVSI (66, 103) Tumour size (100) Lymph node metastasis (99, 103) Recurrence (103)
	High Estriol (E3) High Estrone sufate (E1-S)	Non-myoinvasive tumours, low risk of recurrence and improved overall survival (104) Increased relapse (104)
	
Metabolites	Bradykinin, heme, lactic acid, homocysteine, myristic acid, valine, progesterone, threonine, stearic acid, sarcosine, glycine etc	Associated with histological subtype (13, 105, 106)
	Hydroxysphingomyelins, phospatidylcholines, estrogen metabolites	Associated with deep myometrial invasion (13, 106–108)
	Hexadecadienyl carnitine, phosphatidylcholines	Associated with LVSI (13, 107)
	Spermine, acylcholines, sphingolipids, linoleic acid, myristic acid, polyamines, ceramides	Associated with recurrence (13, 105)
	Methionine sulfoxide	Poor survival (109)
Circulating tumour cells (CTC)	Detection of CTC	Poor progression-free survival (92) Association with non-endometrioid cancer (92) Large tumour size (>5cm) (92) Lymph node involvement (93) Deep myometrial invasion (93)
Circulating tumour DNA (ctDNA)	Presence of ctDNA	Associated with type II tumours (87). Elevated in grades 2 and 3 endometrial cancer (85, 86)
	Serum ctDNA integrity	Elevated in LVSI (85)
	Plasma p53 antibody	Linked to serous tumours (87) Linked to higher grade in Type I tumours (87)
	Plasma *KRAS* mutation	Elevated in grade 2 of type I tumours (87)
	Presence of plasma mutation (*CTNNBI, KRAS, PTEN*, or *PIK3CA*)	Linked to tumour stage (91) Deep myometrial invasion (91) LVSI (91) Large tumour size (91)
Immune/inflammatory parameters	Elevated CRP	Associated with poor overall and cancer-specific survival(65, 80, 81, 110) Stage (111, 112) Lymph node involvement (112)
	Glasgow prognostic score	Survival and recurrence (113)
	Inflammatory parameters (NLR,MLR,PLR,SII etc)	Adverse clinico-pathological features and outcomes (94, 95, 114–117)

## Radiomic Prognostic Profiling of Endometrial Cancer

Radiomic-based risk-stratification models are emerging prognostic systems in endometrial cancer (118). Radiomics deals with the high-throughput mining of quantitative tomographic image parameters and their application in clinical decision making (119). There is growing evidence for the potential utility of radiomic techniques in improving cancer diagnostic, prognostic and predictive accuracy across various tumour sites (118, 119). This has been made possible by the advances in artificial intelligence and machine learning techniques, thus allowing for an in-depth tumour characterisation. Studies have been consistent in suggesting the potential utility of radiomic signatures in endometrial cancer risk-stratification and prediction of outcomes (118, 120–123). Increasingly, radiomics is combined with genomic data (radiogenomics) to aid the prediction of genetic variants including microsatellite instability. Veeraravaghan and colleagues proposed an integrated radiomic-clinical classification algorithm that distinguishes MMR-D endometrial tumours from copy number low and copy number high tumours with an AUC of 0.78 (121). Chen and colleagues found that an MRI-based radiomic model had better discrimination than clinical and conventional MRI parameters in predicting low risk endometrial cancer (124). Yan and colleagues showed that radiomic based models can aid the prediction of pelvic lymph node metastasis in endometrial cancer (120). A high-quality, robust and generalizable radiomic risk-prediction model is dependent on the optimal collection and integration of data from multimodal sources and rigor in model development and implementation (119, 125).

## Clinical Parameters and Endometrial Cancer Prognosis

Several clinical parameters have been associated with endometrial cancer survival outcomes. They include age at diagnosis, body mass index (BMI) and the presence of comorbidities (126). Age at diagnosis is universally accepted as prognostic for most adult cancers, with older patients having worse outcomes. In the UK, endometrial cancer mortality rates were highest in women aged 85 to 89 between 2016 and 2018, with over 50% of all endometrial cancer deaths occurring in those aged 75 and over (3). An important consideration is whether this association is purely related to age or other unfavourable prognostic factors that are associated with age (126). Studies have been consistent in reporting an association between advancing age and the presence of adverse tumour related parameters (127–129). For example, Lachance and colleagues studied 396 women with endometrial cancer and reported a higher prevalence of aggressive disease, specifically higher grade, late stage, non-endometrioid endometrial cancers in those >65 years of age (129). In a retrospective analysis of 551 endometrial cancer patients, Son and colleagues found that age ≤40 years was associated with non-invasive cancers, less lympho-vascular space invasion and a higher body mass index (130). Lee et al, in a study of over 15,000 women with endometrial cancer, reported a higher rate of serous histology in those >40 years and a 5-year disease-specific survival rate of 86.4% compared to 93.2% in women <40 years (127). Following adjustment for histology and adjuvant therapy, the survival disadvantage persisted. Other factors including differential treatment and treatment-related morbidity may be contributory to these trends. Koul and colleagues found that older women (≥75 years) were less likely to be offered adjuvant therapy and had a significantly lower 5-year cancer-specific survival rate compared to those <75 years (128). Zeng and colleagues reported a higher rate of post-operative morbidity in elderly endometrial cancer patients undergoing robotic surgery (131). These findings are consistent with previously published data where age has been reported to independently impact on endometrial cancer outcomes, including risk of recurrence (130, 132–135).

Obesity is the most important modifiable risk factor in endometrial cancer, with every 5kg/m^2^ increase in BMI conferring a 60% increased risk of the disease (136). Obesity-driven endometrial cancers are usually low grade, early stage, endometrioid tumours with a favourable prognosis when compared with the biologically aggressive non-endometrioid endometrial cancer phenotypes (136–138). Despite the survival advantages offered by favourable tumour biology, obesity is associated with higher all-cause mortality due to comorbid health conditions, particularly cardiovascular disease (139). Indeed, cardiovascular disease is the leading cause of death among endometrial cancer survivors (140). Arem and colleagues found that women with BMI ≥35kg/m^2^ had an almost 5-fold higher risk of cardiovascular-related mortality 10 years post diagnosis compared with those with BMI <25kg/m^2^ (141). Secord and colleagues, in a meta-analysis involving 665,694 endometrial cancer cases reported significantly higher odds of all-cause mortality with increasing BMI, with the highest risk for those with class III obesity (BMI≥40kg/m^2^) (139). Obesity may also influence cancer-specific mortality from treatment-related factors (142). As an example, women with class III obesity are less likely to be offered hysterectomy, have a higher risk of perioperative morbidity and are more likely to receive suboptimal doses of chemotherapy from dose capping (142–146). Obesity may also impact on the optimal delivery of adjuvant radiation due to physical, technical and dosimetric constraints, thus contributing to poorer outcomes (147). Whilst obesity certainly impacts on endometrial outcomes, it is unclear whether weight loss interventions can improve survival and work in this space is on-going (148).

Studies have shown that women with a higher Age-adjusted Charlson-Comorbidity (AAC) index scores are at a greater risk of overall mortality, but not cancer-specific mortality or disease recurrence (149). Robbins and colleagues, in an analysis of 671 patients with FIGO stage I-II endometrioid endometrial cancer, report that high AAC scores independently predict short overall survival (149). It remains unclear whether lifestyle changes, including weight loss and dietary modifications, can reduce cardiovascular risk in endometrial cancer survivors, although this is a tantalizing concept our group seeks to explore further. There is growing evidence that thyroid dysfunction may be linked to survival outcomes in endometrial cancer. The small study by Seebacher and colleagues reported poor disease-specific survival in women with TSH>2.5 mU/L (150). Our group recently found that endometrial cancer patients with comorbid hypothyroidism have significantly improved overall, cancer-specific and recurrence-free survival than those who are euthyroid (151). A prospective validation of these findings is warranted and the underlying mechanisms will need to be elucidated prior to clinical translation. Whether type 2 diabetes mellitus (T2DM) status impacts endometrial cancer survival outcomes is unclear. The meta-analysis by Zhang and colleagues involving 12,195 endometrial cancer cases and 575 deaths found no evidence of an association between T2DM status and endometrial cancer mortality (152). A more recent meta-analysis of five cohort studies by Laio and colleagues concluded that the data linking T2DM status and endometrial cancer-specific mortality are inconsistent. This analysis was limited by considerable clinical and methodological heterogeneity of included studies (153). In two of the included studies, a pooled relative risk of 1.32 (95% CI 1.10, 1.60. p=0.003) was reported. One study reported a hazard ratio of 1.64 (95% CI 0.17, 9.60, p=0.58) while the other three studies reported SMRs that could not be quantitatively synthesized (153). Further research is needed to clarify the prognostic impact of T2DM status on endometrial cancer outcomes.

## Sociodemographic Associations With Prognosis

There is good evidence to suggest that ethnicity affects outcomes from endometrial cancer (154, 155). In the USA, Black women are more likely to be diagnosed with late stage disease and biologically aggressive endometrial cancer phenotypes (high grade, non-endometrioid cancers) than women of White ethnicity (154, 156–159). Park and colleagues found that non-Hispanic Black women had significantly shorter overall survival than non-Hispanic White women in an equal access healthcare system, despite correcting for traditional clinico-pathological characteristics, suggesting that other factors including molecular phenotypic differences might be contributing (155). It has been postulated that differential expression of specific tumour markers such as p53, *PTEN*, *HER2/neu* and *PIK3R1* mutations may explain some of the racial disparities (160, 161). *PTEN* mutation portends a favourable prognosis and has been reported to be less common in Black women compared to White women (162). *TP53* mutations, on the other hand, portend an unfavourable prognosis and are more common in Black women (163). Studies have also shown that women of Black ethnicity are less likely to undergo hysterectomy (160, 164) or receive adjuvant therapy than their White counterparts (165, 166). A review of the US National Cancer Database found that 47% of the 19,594 endometrial cancer patients who met the criteria for adjuvant radiation failed to receive radiation. The omission of adjuvant radiation was more common amongst Black, Asian and Hispanic women as well as those of lower socioeconomic status (166). Differences in comorbid conditions may also contribute to racial disparities in outcomes. Studies have been consistent in suggesting a higher comorbidity burden amongst Black women compared to women of White ethnicity (167, 168). Tarney and colleagues found that Black women <65 years with endometrial cancer are more likely to die from non-cancer related causes than White women (169).

Socioeconomic status has been linked with endometrial cancer outcomes too. Factors such as differential access to health care, level of income, educational status and areal-level economic deprivation may be contributory. Bedir and colleagues analyzed data on 21,602 German women with endometrial cancer and found differences in survival according to district level socioeconomic deprivation (170). In a Swedish study, women from the higher social groups were less likely to be diagnosed with advanced stage disease and non-endometrioid cancers, and had more favourable outcomes than women from the lower social groups (171). These findings are consistent with those reported in several high-income countries (149, 164, 172, 173). In the UK, results have been conflicting (174–176). Donkers and colleagues found no evidence of a socioeconomic disparity in survival after adjusting for confounding factors (175). Using the English multiple indices of deprivation, Njoku and colleagues found that women from more deprived neighbourhoods were more likely to present with fatal recurrence than those from less deprived areas (176). Further research is needed to confirm these findings and identify modifiable contributing factors.

## Conclusion

Several clinical, sociodemographic and tumour specific parameters have emerged as important endometrial cancer prognostic biomarkers. The Cancer Genome Atlas and subsequent clinically translatable molecular classification systems, in particular, hold great promise to refine current endometrial cancer risk stratification systems. The clinical utility of endometrial cancer molecular classification in guiding adjuvant therapy and recurrence monitoring is yet to be defined and must now be prioritised. Blood-based markers including systemic inflammatory parameters, proteins and metabolites, and circulating tumour cells have also shown potential to refine endometrial cancer risk stratification algorithms and their prospective validation in larger study cohorts is warranted. The impact of socioeconomic status and ethnicity on endometrial cancer outcomes is becoming more apparent and studies exploring the factors underlying these disparities are urgently needed.

## Author Contributions

Conceptualization- KN and EJC. Writing- original draft preparation KN. Writing- review and editing KN, CEB, and EJC. Supervision- EJC. Funding acquisition- EJC. All authors read and approved the final version for submission.

## Funding

KN is supported by Cancer Research UK (CRUK) Manchester Cancer Research Centre Clinical Research Fellowship (C147/A25254) and the Wellcome Trust Manchester Translational Informatics Training Scheme. CEB is supported by Manchester University NHS Foundation Trust Clinical Research Fellowship, EJC is supported by a National Institute for Health Research (NIHR) Advanced Fellowship (NIHR300650) and the Manchester NIHR Biomedical Research Centre (IS-BRC-1215-20007).

## Conflict of Interest

The authors declare that the research was conducted in the absence of any commercial or financial relationships that could be construed as a potential conflict of interest.

## Publisher’s Note

All claims expressed in this article are solely those of the authors and do not necessarily represent those of their affiliated organizations, or those of the publisher, the editors and the reviewers. Any product that may be evaluated in this article, or claim that may be made by its manufacturer, is not guaranteed or endorsed by the publisher.

## References

[1] Sung H Ferlay J Siegel RL Laversanne M Soerjomataram I Jemal A. Global Cancer Statistics 2020: GLOBOCAN Estimates of Incidence and Mortality Worldwide for 36 Cancers in 185 Countries. CA Cancer J Clin (2021) 71(3):209–49. doi: 10.3322/caac.21660 33538338

[2] Crosbie E Morrison J. The Emerging Epidemic of Endometrial Cancer: Time to Take Action. Cochrane Database Syst Rev (2014) 12:1465–858. doi: 10.1002/14651858.ED000095 PMC10845858 25549990

[3] CRUK. Uterine Cancer Incidence Statistics. CRUK (2020). CRUK. Available at: www.cancerresearchuk.org; http://www.cancerresearchuk.org/health-professional/cancer-statistics/statistics-by-cancer-type/uterine-cancer/incidence#heading-One (Accessed 1/6/2020).

[4] Morice P Leary A Creutzberg C Abu-Rustum N Darai E. Endometrial Cancer. Lancet (2016) 387(10023):1094–108. doi: 10.1016/S0140-6736(15)00130-0 26354523

[5] Ryan NAJ McMahon R Tobi S Snowsill T Esquibel S Wallace AJ. The Proportion of Endometrial Tumours Associated With Lynch Syndrome (PETALS): A Prospective Cross-Sectional Study. PloS Med (2020) 17(9):e1003263. doi: 10.1371/journal.pmed.1003263 32941469 PMC7497985

[6] Njoku K Abiola J Russell J Crosbie EJ. Endometrial Cancer Prevention in High-Risk Women. Best Pract Res Clin Obstet Gynaecol (2020) 65:66–78. doi: 10.1016/j.bpobgyn.2019.12.005 32107136

[7] Jones ER O’Flynn H Njoku K Crosbie EJ. Detecting Endometrial Cancer. Obstet Gynaecol (2021) 23(2):103–12. doi: 10.1111/tog.12722

[8] Colombo N Creutzberg C Amant F Bosse T González-Martín A Ledermann J. ESMO-ESGO-ESTRO Consensus Conference on Endometrial Cancer: Diagnosis, Treatment and Follow-Up. Int J Gynecol Cancer (2016) 26(1):2–30. doi: 10.1097/IGC.0000000000000609 26645990 PMC4679344

[9] McAlpine JN Temkin SM Mackay HJ. Endometrial Cancer: Not Your Grandmother’s Cancer. Cancer (2016) 122(18):2787–98. doi: 10.1002/cncr.30094 27308732

[10] Vermij L Smit V Nout R Bosse T. Incorporation of Molecular Characteristics Into Endometrial Cancer Management. Histopathology (2020) 76(1):52–63. doi: 10.1111/his.14015 31846532 PMC6972558

[11] Sechidis K Papangelou K Metcalfe PD Svensson D Weatherall J Brown G. Distinguishing Prognostic and Predictive Biomarkers: An Information Theoretic Approach. Bioinformatics (2018) 34(19):3365–76. doi: 10.1093/bioinformatics/bty357 PMC6157098 29726967

[12] Group F-NBW. BEST (Biomarkers, EndpointS, and Other Tools) Resource [Internet]. Silver Spring (MD): Food and Drug Administration (US) (2018). 2016-Co-published by National Institutes of Health (US), Bethesda (MD).

[13] Njoku K Sutton CJ Whetton AD Crosbie EJ. Metabolomic Biomarkers for Detection, Prognosis and Identifying Recurrence in Endometrial Cancer. Metabolites (2020) 10(8):314. doi: 10.3390/metabo10080314 PMC7463916 32751940

[14] Bokhman JV. Two Pathogenetic Types of Endometrial Carcinoma. Gynecol Oncol (1983) 15(1):10–7. doi: 10.1016/0090-8258(83)90111-7 6822361

[15] Murali R Soslow RA Weigelt B. Classification of Endometrial Carcinoma: More Than Two Types. Lancet Oncol (2014) 15(7):e268–78. doi: 10.1016/S1470-2045(13)70591-6 24872110

[16] Amant F Moerman P Neven P Timmerman D Van Limbergen E Vergote I. Endometrial Cancer. Lancet (2005) 366(9484):491–505. doi: 10.1016/S0140-6736(05)67063-8 16084259

[17] Morrison J Balega J Buckley L Clamp A Crosbie E Drew Y. British Gynaecological Cancer Society (BGCS) Uterine Cancer Guidelines: Recommendations for Practice. Eur J Obstet Gynecol Reprod Biol (2022) 270:50–89. doi: 10.1016/j.ejogrb.2021.11.423 35065448

[18] Setiawan VW Yang HP Pike MC McCann SE Yu H Xiang Y-B. Type I and II Endometrial Cancers: Have They Different Risk Factors? J Clin Oncol (2013) 31(20):2607. doi: 10.1200/jco.2012.48.2596 23733771 PMC3699726

[19] Dedes KJ Wetterskog D Ashworth A Kaye SB Reis-Filho JS. Emerging Therapeutic Targets in Endometrial Cancer. Nat Rev Clin Oncol (2011) 8(5):261–71. doi: 10.1038/nrclinonc.2010.216 21221135

[20] Urick ME Bell DW. Clinical Actionability of Molecular Targets in Endometrial Cancer. Nat Rev Cancer (2019) 19(9):510–21. doi: 10.1038/s41568-019-0177-x PMC7446243 31388127

[21] Pecorelli S. Revised FIGO Staging for Carcinoma of the Vulva, Cervix, and Endometrium. Int J Gynecol Obstet (2009) 105(2):103–4. doi: 10.1016/j.ijgo.2009.02.012 19367689

[22] Soslow RA Tornos C Park KJ Malpica A Matias-Guiu X Oliva E. Endometrial Carcinoma Diagnosis: Use of FIGO Grading and Genomic Subcategories in Clinical Practice: Recommendations of the International Society of Gynecological Pathologists. Int J Gynecol Pathol (2019) 38(1 Suppl 1):S64. doi: 10.1097/PGP.0000000000000518 30550484 PMC6295928

[23] Uharček P. Prognostic Factors in Endometrial Carcinoma. J Obstet Gynaecol Res (2008) 34(5):776–83. doi: 10.1111/j.1447-0756.2008.00796.x 18958927

[24] Wang J Xu P Yang X Yu Q Xu X Zou G. Association of Myometrial Invasion With Lymphovascular Space Invasion, Lymph Node Metastasis, Recurrence, and Overall Survival in Endometrial Cancer: A Meta-Analysis of 79 Studies With 68,870 Patients. Front Oncol (2021) 11:762329. doi: 10.3389/fonc.2021.762329 34746002 PMC8567142

[25] Moatasim A Hameed Z Ahmad I. Assessment of Lymphovascular Invasion in Early Stage Endometrial Carcinoma-a Retrospective Study. Surg Exp Pathol (2021) 4(1):1–6. doi: 10.1186/s42047-021-00091-6

[26] Cusano E Myers V Samant R Sudai T Keller A Le T. Prognostic Significance of Lymphovascular Space Invasion in the Absence of Lymph Node Metastases in Early-Stage Endometrial Cancer. Int J Gynecol Cancer (2018) 28(5):890–94. doi: 10.1097/IGC.0000000000001229 29538248

[27] Concin N Matias-Guiu X Vergote I Cibula D Mirza MR Marnitz S. ESGO/ESTRO/ESP Guidelines for the Management of Patients With Endometrial Carcinoma. Int J Gynecol Cancer (2021) 31(1):12–39. doi: 10.1136/ijgc-2020-002230 33397713

[28] Bendifallah S Canlorbe G Collinet P Arsene E Huguet F Coutant C. Just How Accurate Are the Major Risk Stratification Systems for Early-Stage Endometrial Cancer? Br J Cancer (2015) 112(5):793–801. doi: 10.1038/bjc.2015.35 25675149 PMC4453957

[29] Kommoss S McConechy MK Kommoss F Leung S Bunz A Magrill J. Final Validation of the ProMisE Molecular Classifier for Endometrial Carcinoma in a Large Population-Based Case Series. Ann Oncol (2018) 29(5):1180–8. doi: 10.1093/annonc/mdy058 29432521

[30] Gilks CB Oliva E Soslow RA. Poor Interobserver Reproducibility in the Diagnosis of High-Grade Endometrial Carcinoma. Am J Surg Pathol (2013) 37(6):874–81. doi: 10.1097/PAS.0b013e31827f576a 23629444

[31] Thomas S Hussein Y Bandyopadhyay S Cote M Hassan O Abdulfatah E. Interobserver Variability in the Diagnosis of Uterine High-Grade Endometrioid Carcinoma. Arch Pathol Lab Med (2016) 140(8):836–43. doi: 10.5858/arpa.2015-0220-OA PMC5656271 27139150

[32] De Boer SM Wortman BG Bosse T Powell ME Singh N Hollema H. Clinical Consequences of Upfront Pathology Review in the Randomised PORTEC-3 Trial for High-Risk Endometrial Cancer. Ann Oncol (2018) 29(2):424–30. doi: 10.1093/annonc/mdx753 PMC5834053 29190319

[33] Jamieson A Bosse T McAlpine JN. The Emerging Role of Molecular Pathology in Directing the Systemic Treatment of Endometrial Cancer. Ther Adv Med Oncol (2021) 13:17588359211035960. doi: 10.1177/17588359211035959 PMC8366203 34408794

[34] Levine DA Network CGAR. Integrated Genomic Characterization of Endometrial Carcinoma. Nature (2013) 497(7447):67–73. doi: 10.1038/nature12113 23636398 PMC3704730

[35] Vicky M MacKay H Ray-Coquard I Levine DA Westin SN Daisuke A. Endometrial Cancer (Primer). Nat Rev Dis Primers (2021) 7(1):88. doi: 10.1038/s41572-021-00324-8 34887451 PMC9421940

[36] McAlpine J Leon-Castillo A Bosse T. The Rise of a Novel Classification System for Endometrial Carcinoma; Integration of Molecular Subclasses. J Pathol (2018) 244(5):538–49. doi: 10.1002/path.5034 29344951

[37] Stubert J Gerber B. Current Issues in the Diagnosis and Treatment of Endometrial Carcinoma. Geburtshilfe Frauenheilkd (2016) 76(02):170–5. doi: 10.1055/s-0035-1558230 PMC4771503 26941450

[38] Raffone A Travaglino A Gabrielli O Micheli M Zuccalà V Bitonti G. Clinical Features of ProMisE Groups Identify Different Phenotypes of Patients With Endometrial Cancer. Arch Gynecol Obstet (2021) 303(6):1393–400. doi: 10.1007/s00404-021-06028-4 PMC8087601 33754186

[39] Bosse T Powell M Crosbie E Leary A Kroep J Han K. 595 Implementation of Collaborative Translational Research (TransPORTEC) Findings in an International Endometrial Cancer Clinical Trials Program (RAINBO). BMJ Spec J (2021) 31:A108–9. doi: 10.1136/ijgc-2021-ESGO.171

[40] Talhouk A McConechy MK Leung S Li-Chang HH Kwon JS Melnyk N. A Clinically Applicable Molecular-Based Classification for Endometrial Cancers. Br J Cancer (2015) 113(2):299–310. doi: 10.1038/bjc.2015.190 26172027 PMC4506381

[41] Raffone A Travaglino A Mascolo M Carbone L Guida M Insabato L. TCGA Molecular Groups of Endometrial Cancer: Pooled Data About Prognosis. Gynecol Oncol (2019) 155(2):374–83. doi: 10.1016/j.ygyno.2019.08.019 31472940

[42] Kuhn E Bahadirli-Talbott A Shih I-M. Frequent CCNE1 Amplification in Endometrial Intraepithelial Carcinoma and Uterine Serous Carcinoma. Mod Pathol (2014) 27(7):1014–9. doi: 10.1038/modpathol.2013.209 24309323

[43] Fader AN Roque DM Siegel E Buza N Hui P Abdelghany O. Randomized Phase II Trial of Carboplatin–Paclitaxel Compared With Carboplatin–Paclitaxel–Trastuzumab in Advanced (Stage III–IV) or Recurrent Uterine Serous Carcinomas That Overexpress Her2/Neu (NCT01367002): Updated Overall Survival Analysis. Clin Cancer Res (2020) 26(15):3928–35. doi: 10.1158/1078-0432.CCR-20-0953 PMC8792803 32601075

[44] Kurnit KC Kim GN Fellman BM Urbauer DL Mills GB Zhang W. CTNNB1 (Beta-Catenin) Mutation Identifies Low Grade, Early Stage Endometrial Cancer Patients at Increased Risk of Recurrence. Mod Pathol (2017) 30(7):1032–41. doi: 10.1038/modpathol.2017.15 PMC5493522 28281553

[45] Depreeuw J Stelloo E Osse EM Creutzberg CL Nout RA Moisse M. Amplification of 1q32. 1 Refines the Molecular Classification of Endometrial Carcinoma. Clin Cancer Res (2017) 23(23):7232–41. doi: 10.1158/1078-0432.CCR-17-0566 28939739

[46] McConechy MK Talhouk A Leung S Chiu D Yang W Senz J. Endometrial Carcinomas With POLE Exonuclease Domain Mutations Have a Favorable Prognosis. Clin Cancer Res (2016) 22(12):2865–73. doi: 10.1158/1078-0432.CCR-15-2233 26763250

[47] Dou Y Kawaler EA Zhou DC Gritsenko MA Huang C Blumenberg L. Proteogenomic Characterization of Endometrial Carcinoma. Cell (2020) 180(4):729–48. doi: 10.1158/1538-7445.AM2020-6580 PMC7233456 32059776

[48] Stelloo E Bosse T Nout RA MacKay HJ Church DN Nijman HW. Refining Prognosis and Identifying Targetable Pathways for High-Risk Endometrial Cancer; a TransPORTEC Initiative. Mod Pathol (2015) 28(6):836–44. doi: 10.1038/modpathol.2015.43 25720322

[49] Talhouk A McConechy MK Leung S Yang W Lum A Senz J. Confirmation of ProMisE: A Simple, Genomics-Based Clinical Classifier for Endometrial Cancer. Cancer (2017) 123(5):802–13. doi: 10.1002/cncr.30496 28061006

[50] Stelloo E Nout RA Naves LCLM Ter Haar NT Creutzberg CL Smit VT. High Concordance of Molecular Tumor Alterations Between Pre-Operative Curettage and Hysterectomy Specimens in Patients With Endometrial Carcinoma. Gynecol Oncol (2014) 133(2):197–204. doi: 10.1016/j.ygyno.2014.02.012 24556061

[51] Talhouk A Hoang LN McConechy MK Nakonechny Q Leo J Cheng A. Molecular Classification of Endometrial Carcinoma on Diagnostic Specimens Is Highly Concordant With Final Hysterectomy: Earlier Prognostic Information to Guide Treatment. Gynecol Oncol (2016) 143(1):46–53. doi: 10.1016/j.ygyno.2016.07.090 27421752 PMC5521211

[52] Batista TP Cavalcanti CLC Tejo AAG Bezerra ALR. Accuracy of Preoperative Endometrial Sampling Diagnosis for Predicting the Final Pathology Grading in Uterine Endometrioid Carcinoma. Eur J Surg Oncol (2016) 42(9):1367–71. doi: 10.1016/j.ejso.2016.03.009 27052799

[53] Kim SR Cloutier BT Leung S Cochrane D Britton H Pina A. Molecular Subtypes of Clear Cell Carcinoma of the Endometrium: Opportunities for Prognostic and Predictive Stratification. Gynecol Oncol (2020) 158(1):3–11. doi: 10.1016/j.ygyno.2020.04.043 32331700

[54] DeLair DF Burke KA Selenica P Lim RS Scott SN Middha S. The Genetic Landscape of Endometrial Clear Cell Carcinomas. J Pathol (2017) 243(2):230–41. doi: 10.1002/path.4947 PMC5708127 28718916

[55] Bosse T Nout RA McAlpine JN McConechy MK Britton H Hussein Y. Molecular Classification of Grade 3 Endometrioid Endometrial Cancers Identifies Distinct Prognostic Subgroups. Am J Surg Pathol (2018) 42(5):561. doi: 10.1097/PAS.0000000000001020 29505428 PMC5893364

[56] Brett MA Atenafu EG Singh N Ghatage P Clarke BA Nelson GS. Equivalent Survival of P53 Mutated Endometrial Endometrioid Carcinoma Grade 3 and Endometrial Serous Carcinoma. Int J Gynecol Pathol (2021) 40(2):116–23. doi: 10.1097/PGP.0000000000000674 32265358

[57] Stelloo E Nout RA Osse EM Jürgenliemk-Schulz IJ Jobsen JJ Lutgens LC. Improved Risk Assessment by Integrating Molecular and Clinicopathological Factors in Early-Stage Endometrial Cancer—Combined Analysis of the PORTEC Cohorts. Clin Cancer Res (2016) 22(16):4215–24. doi: 10.1158/1078-0432.CCR-15-2878 27006490

[58] Zeimet AG Reimer D Huszar M Winterhoff B Puistola U Abdel Azim S. L1CAM in Early-Stage Type I Endometrial Cancer: Results of a Large Multicenter Evaluation. J Natl Cancer Inst (2013) 105(15):1142–50. doi: 10.1093/jnci/djt144 23781004

[59] Van Der Putten LJM Visser N Van De Vijver K Santacana M Bronsert P Bulten J. L1CAM Expression in Endometrial Carcinomas: An ENITEC Collaboration Study. Br J Cancer (2016) 115(6):716–24. doi: 10.1038/bjc.2016.235 PMC5023774 27505134

[60] Hua T Liu S Xin X Jin Z Liu Q Chi S. Prognostic Significance of L1 Cell Adhesion Molecule in Cancer Patients: A Systematic Review and Meta-Analysis. Oncotarget (2016) 7(51):85196. doi: 10.18632/oncotarget.13236 27833079 PMC5356729

[61] Visser N Bulten J van der Wurff AAM Boss EA Bronkhorst CM Feijen HWH. PIpelle Prospective ENDOmetrial Carcinoma (PIPENDO) Study, Pre-Operative Recognition of High Risk Endometrial Carcinoma: A Multicentre Prospective Cohort Study. BMC Cancer (2015) 15(1):1–6. doi: 10.1186/s12885-015-1487-3 26123742 PMC4485884

[62] Trovik J Wik E Werner HMJ Krakstad C Helland H Vandenput I. Hormone Receptor Loss in Endometrial Carcinoma Curettage Predicts Lymph Node Metastasis and Poor Outcome in Prospective Multicentre Trial. Eur J Cancer (2013) 49(16):3431–41. doi: 10.1016/j.ejca.2013.06.016 23932335

[63] Morelli M Scumaci D Di Cello A Venturella R Donato G Faniello MC. DJ-1 in Endometrial Cancer: A Possible Biomarker to Improve Differential Diagnosis Between Subtypes. Int J Gynecol Cancer (2014) 24(4):649–58. doi: 10.1097/IGC.0000000000000102 24614826

[64] Reijnen C Gogou E Visser NCM Engerud H Ramjith J van der Putten LJM. Preoperative Risk Stratification in Endometrial Cancer (ENDORISK) by a Bayesian Network Model: A Development and Validation Study. PloS Med (2020) 17(5):e1003111. doi: 10.1371/journal.pmed.1003111 32413043 PMC7228042

[65] Weinberger V Bednarikova M Hausnerova J Ovesna P Vinklerova P Minar L. A Novel Approach to Preoperative Risk Stratification in Endometrial Cancer: The Added Value of Immunohistochemical Markers. Front Oncol (2019) 9:265. doi: 10.3389/fonc.2019.00265 31032226 PMC6473394

[66] Ravegnini G De Leo A Coada C Gorini F de Biase D Ceccarelli C. Identification of MiR-499a-5p as a Potential Novel Biomarker for Risk Stratification in Endometrial Cancer. Front Oncol (2021) 4321. doi: 10.3389/fonc.2021.757678 PMC8597024 34804952

[67] León-Castillo A de Boer SM Powell ME Mileshkin LR Mackay HJ Leary A. Molecular Classification of the PORTEC-3 Trial for High-Risk Endometrial Cancer: Impact on Prognosis and Benefit From Adjuvant Therapy. J Clin Oncol (2020) 38(29):3388–97. doi: 10.1200/JCO.20.00549 PMC7527156 32749941

[68] Rimel BJ. A Randomized, Phase II Study Comparing Single-Agent Olaparib, Single Agent Cediranib, and the Combination of Cediranib/Olaparib in Women With Recurrent, Persistent or Metastatic Endometrial Cancer. Gynecol Oncol (2021) 162:S43–4. doi: 10.1016/S0090-8258(21)00727-7 PMC11168450 38127487

[69] Talhouk A McAlpine JN. New Classification of Endometrial Cancers: The Development and Potential Applications of Genomic-Based Classification in Research and Clinical Care. Gynecol Oncol Res Pract (2016) 3(1):1–12. doi: 10.1186/s40661-016-0035-4 27999680 PMC5154099

[70] Marabelle A Le DT Ascierto PA Di Giacomo AM De Jesus-Acosta A Delord J-P. Efficacy of Pembrolizumab in Patients With Noncolorectal High Microsatellite Instability/Mismatch Repair–Deficient Cancer: Results From the Phase II KEYNOTE-158 Study. J Clin Oncol (2020) 38(1):1. doi: 10.1200/JCO.19.02105 31682550 PMC8184060

[71] Oaknin A Tinker AV Gilbert L Samouëlian V Mathews C Brown J. Clinical Activity and Safety of the Anti–Programmed Death 1 Monoclonal Antibody Dostarlimab for Patients With Recurrent or Advanced Mismatch Repair–Deficient Endometrial Cancer: A Nonrandomized Phase 1 Clinical Trial. JAMA Oncol (2020) 6(11):1766–72. doi: 10.1001/jamaoncol.2020.4515 PMC7530821 33001143

[72] Marabelle A Fakih M Lopez J Shah M Shapira-Frommer R Nakagawa K. Association of Tumour Mutational Burden With Outcomes in Patients With Advanced Solid Tumours Treated With Pembrolizumab: Prospective Biomarker Analysis of the Multicohort, Open-Label, Phase 2 KEYNOTE-158 Study. Lancet Oncol (2020) 21(10):1353–65. doi: 10.1016/S1470-2045(20)30445-9 32919526

[73] Wan YL Beverley-Stevenson R Carlisle D Clarke S Edmondson RJ Glover S. Working Together to Shape the Endometrial Cancer Research Agenda: The Top Ten Unanswered Research Questions. Gynecol Oncol (2016) 143(2):287–93. doi: 10.1016/j.ygyno.2016.08.333 27593736

[74] Behrouzi R Barr CE Crosbie EJ. HE4 as a Biomarker for Endometrial Cancer. Cancers (2021) 13(19):4764. doi: 10.3390/cancers13194764 34638250 PMC8507549

[75] Coll-de la Rubia E Martinez-Garcia E Dittmar G Gil-Moreno A Cabrera S Colas E. Prognostic Biomarkers in Endometrial Cancer: A Systematic Review and Meta-Analysis. J Clin Med (2020) 9(6):1900. doi: 10.3390/jcm9061900 PMC7356541 32560580

[76] Njoku K Chiasserini D Whetton AD Crosbie EJ. Proteomic Biomarkers for the Detection of Endometrial Cancer. Cancers (Basel) (2019) 11(10):1572. doi: 10.3390/cancers11101572 PMC6826703 31623106

[77] Niloff JM Klug TL Schaetzl E Zurawski VR Jr Knapp RC Bast RC Jr. Elevation of Serum CA125 in Carcinomas of the Fallopian Tube, Endometrium, and Endocervix. Am J Obstet Gynecol (1984) 148(8):1057–8. doi: 10.1016/S0002-9378(84)90444-7 6201072

[78] Kurihara T Mizunuma H Obara M Andoh K Ibuki Y Nishimura T. Determination of a Normal Level of Serum CA125 in Postmenopausal Women as a Tool for Preoperative Evaluation and Postoperative Surveillance of Endometrial Carcinoma. Gynecol Oncol (1998) 69(3):192–6. doi: 10.1006/gyno.1998.5018 9648586

[79] Sood AK Buller RE Burger RA Dawson JD Sorosky JI Berman M. Value of Preoperative CA 125 Level in the Management of Uterine Cancer and Prediction of Clinical Outcome. Obstet Gynecol (1997) 90(3):441–7. doi: 10.1016/S0029-7844(97)00286-X 9277659

[80] Dotters DJ. Preoperative CA 125 in Endometrial Cancer: Is It Useful? Am J Obstet Gynecol (2000) 182(6):1328–34. doi: 10.1067/mob.2000.106251 10871446

[81] Yildiz A Yetimalar H Kasap B Aydin C Tatar S Soylu F. Preoperative Serum CA 125 Level in the Prediction of the Stage of Disease in Endometrial Carcinoma. Eur J Obstet Gynecol Reprod Biol (2012) 164(2):191–5. doi: 10.1016/j.ejogrb.2012.05.038 22727919

[82] Han S Lee SH Kim DH Kim JW Park N Kang S. Evaluation of Preoperative Criteria Used to Predict Lymph Node Metastasis in Endometrial Cancer. Acta Obstet Gynecol Scand (2010) 89(2):168–74. doi: 10.3109/00016340903370114 19916890

[83] Jiang T Huang L Zhang S. Preoperative Serum CA125: A Useful Marker for Surgical Management of Endometrial Cancer. BMC Cancer (2015) 15(1):1–8. doi: 10.1186/s12885-015-1260-7 25964114 PMC4438478

[84] Dai C Zheng Y Li Y Tian T Wang M Xu P. Prognostic Values of HE4 Expression in Patients With Cancer: A Meta-Analysis. Cancer Manag Res (2018) 10:4491. doi: 10.2147/CMAR.S178345 30349381 PMC6188164

[85] Vizza E Corrado G De Angeli M Carosi M Mancini E Baiocco E. Serum DNA Integrity Index as a Potential Molecular Biomarker in Endometrial Cancer. J Exp Clin Cancer Res (2018) 37(1):1–9. doi: 10.1186/s13046-018-0688-4 29382392 PMC5791183

[86] Cicchillitti L Corrado G De Angeli M Mancini E Baiocco E Patrizi L. Circulating Cell-Free DNA Content as Blood Based Biomarker in Endometrial Cancer. Oncotarget (2017) 8(70):115230. doi: 10.18632/oncotarget.23247 29383155 PMC5777767

[87] Dobrzycka B Terlikowski SJ Mazurek A Kowalczuk O Niklinska W Chyczewski L. Circulating free DNA, P53 Antibody and Mutations of KRAS Gene in Endometrial Cancer. Int J Cancer (2010) 127(3):612–21. doi: 10.1002/ijc.25077 19960433

[88] Moss EL Gorsia DN Collins A Sandhu P Foreman N Gore A. Utility of Circulating Tumor DNA for Detection and Monitoring of Endometrial Cancer Recurrence and Progression. Cancers (Basel) (2020) 12(8):2231. doi: 10.3390/cancers12082231 PMC7463944 32785174

[89] Tanaka H Tsuda H Nishimura S Nomura H Kataoka F Chiyoda T. Role of Circulating Free Alu DNA in Endometrial Cancer. Int J Gynecol Cancer (2012) 22(1):82–6. doi: 10.1097/IGC.0b013e3182328c94 22146763

[90] Pereira E Camacho-Vanegas O Anand S Sebra R Catalina Camacho S Garnar-Wortzel L. Personalized Circulating Tumor DNA Biomarkers Dynamically Predict Treatment Response and Survival in Gynecologic Cancers. PloS One (2015) 10(12):e0145754. doi: 10.1371/journal.pone.0145754 26717006 PMC4696808

[91] Bolivar AM Luthra R Mehrotra M Chen W Barkoh BA Hu P. Targeted Next-Generation Sequencing of Endometrial Cancer and Matched Circulating Tumor DNA: Identification of Plasma-Based, Tumor-Associated Mutations in Early Stage Patients. Mod Pathol (2019) 32(3):405–14. doi: 10.1038/s41379-018-0158-8 PMC6395490 30315273

[92] Lemech CR Ensell L Paterson JC Eminowicz G Lowe H Arora R. Enumeration and Molecular Characterisation of Circulating Tumour Cells in Endometrial Cancer. Oncology (2016) 91(1):48–54. doi: 10.1159/000445999 27256106

[93] Bogani G Liu MC Dowdy SC Cliby WA Kerr SE Kalli KR. Detection of Circulating Tumor Cells in High-Risk Endometrial Cancer. Anticancer Res (2015) 35(2):683–7. 25667446

[94] Ni L Tao J Xu J Yuan X Long Y Yu N. Prognostic Values of Pretreatment Neutrophil-to-Lymphocyte and Platelet-to-Lymphocyte Ratios in Endometrial Cancer: A Systematic Review and Meta-Analysis. Arch Gynecol Obstet (2020) 301(1):251–61. doi: 10.1007/s00404-019-05372-w PMC7028808 31768743

[95] Njoku K Ramchander NC Wan YL Barr CE Crosbie EJ. Pre-Treatment Inflammatory Parameters Predict Survival From Endometrial Cancer: A Prospective Database Analysis. Gynecol Oncol (2021) 164(2022):146–53.. doi: 10.1016/j.ygyno.2021.11.009 PMC8802781 34802721

[96] Reijnen C Visser NCM Kasius JC Boll D Geomini PM Ngo H. Improved Preoperative Risk Stratification With CA-125 in Low-Grade Endometrial Cancer: A Multicenter Prospective Cohort Study. J Gynecol Oncol (2019) 30(5). doi: 10.3802/jgo.2019.30.e70 PMC6658593 31328454

[97] Huang GS Chiu LG Gebb JS Gunter MJ Sukumvanich P Goldberg GL. Serum CA125 Predicts Extrauterine Disease and Survival in Uterine Carcinosarcoma. Gynecol Oncol (2007) 107(3):513–7. doi: 10.1016/j.ygyno.2007.08.060 PMC2696225 17935762

[98] Chen Y Huang C Chien T Huang S Wu C Ho C. Value of Pre-Operative Serum CA125 Level for Prediction of Prognosis in Patients With Endometrial Cancer. Aust N Z J Obstet Gynaecol (2011) 51(5):397–402. doi: 10.1111/j.1479-828X.2011.01325.x 21806586

[99] Panyavaranant P Manchana T. Preoperative Markers for the Prediction of High-Risk Features in Endometrial Cancer. World J Clin Oncol (2020) 11(6):378. doi: 10.5306/wjco.v11.i6.378 32874951 PMC7450819

[100] Espiau Romera A Cuesta Guardiola T Benito Vielba M De Bonrostro Torralba C Coronado Martín PJ Baquedano Mainar L. HE 4 Tumor Marker as a Predictive Factor for Lymphatic Metastasis in Endometrial Cancer. Int J Gynecol Obstet (2020) 149(3):265–8. doi: 10.1002/ijgo.13140 32147821

[101] Kemik P Saatli B Yıldırım N Kemik VD Deveci B Terek MC. Diagnostic and Prognostic Values of Preoperative Serum Levels of YKL-40, HE-4 and DKK-3 in Endometrial Cancer. Gynecol Oncol (2016) 140(1):64–9. doi: 10.1016/j.ygyno.2015.11.020 26607777

[102] Stiekema A Lok CAR Korse CM van Driel WJ van der Noort V Kenter GG. Serum HE4 is Correlated to Prognostic Factors and Survival in Patients With Endometrial Cancer. Virchows Arch (2017) 470(6):655–64. doi: 10.1007/s00428-017-2115-1 28401338

[103] Abbink K Zusterzeel PLM Geurts-Moespot AJ van Herwaarden AE Pijnenborg JMA Sweep FCGJ. HE4 is Superior to CA125 in the Detection of Recurrent Disease in High-Risk Endometrial Cancer Patients. Tumor Biol (2018) 40(2):1010428318757103. doi: 10.1177/1010428318757103 29463191

[104] Audet-Delage Y Grégoire J Caron P Turcotte V Plante M Ayotte P. Estradiol Metabolites as Biomarkers of Endometrial Cancer Prognosis After Surgery. J Steroid Biochem Mol Biol (2018) 178:45–54. doi: 10.1016/j.jsbmb.2017.10.021 29092787

[105] Audet-Delage Y Villeneuve L Grégoire J Plante M Guillemette C. Identification of Metabolomic Biomarkers for Endometrial Cancer and its Recurrence After Surgery in Postmenopausal Women. Front Endocrinol (Lausanne) (2018) 9:87. doi: 10.3389/fendo.2018.00087 29593653 PMC5857535

[106] Raffone A Troisi J Boccia D Travaglino A Capuano G Insabato L. Metabolomics in Endometrial Cancer Diagnosis: A Systematic Review. Acta Obstet Gynecol Scand (2020) 99(9):1135–46. doi: 10.1111/aogs.13847 32180221

[107] Knific T Vouk K Smrkolj Š Prehn C Adamski J Rižner TL. Models Including Plasma Levels of Sphingomyelins and Phosphatidylcholines as Diagnostic and Prognostic Biomarkers of Endometrial Cancer. J Steroid Biochem Mol Biol (2018) 178:312–21. doi: 10.1016/j.jsbmb.2018.01.012 29360580

[108] Audet-Walsh E Lepine J Gregoire J Plante M Caron P Teˆtu B. Profiling of Endogenous Estrogens, Their Precursors, and Metabolites in Endometrial Cancer Patients: Association With Risk and Relationship to Clinical Characteristics. J Clin Endocrinol Metab (2011) 96(2):E330–9. doi: 10.1210/jc.2010-2050 21147881

[109] Strand E Tangen IL Fasmer KE Jacob H Halle MK Hoivik EA. Blood Metabolites Associate With Prognosis in Endometrial Cancer. Metabolites (2019) 9(12):302. doi: 10.3390/metabo9120302 PMC6949989 31847385

[110] Terlikowska KM Dobrzycka B Terlikowski R Sienkiewicz A Kinalski M Terlikowski SJ. Clinical Value of Selected Markers of Angiogenesis, Inflammation, Insulin Resistance and Obesity in Type 1 Endometrial Cancer. BMC Cancer (2020) 20(1):1–10. doi: 10.1186/s12885-020-07415-x PMC7519537 32977765

[111] Schmid M Schneitter A Hinterberger S Seeber J Reinthaller A Hefler L. Association of Elevated C-Reactive Protein Levels With an Impaired Prognosis in Patients With Surgically Treated Endometrial Cancer. Obstet Gynecol (2007) 110(6):1231–6. doi: 10.1097/01.AOG.0000292085.50987.f2 18055714

[112] Wang LJ Zhou H Lu HW Li J Lin ZQ. Prognostic Value of Preoperative Serum High Sensitivity C-Reactive Protein in Patients With Endometrial Cancer. Zhonghua Yi Xue Za Zhi (2011) 91(41):2927–30. 22333616

[113] Saijo M Nakamura K Masuyama H Ida N Haruma T Kusumoto T. Glasgow Prognostic Score Is a Prognosis Predictor for Patients With Endometrial Cancer. Eur J Obstet Gynecol Reprod Biol (2017) 210:355–9. doi: 10.1016/j.ejogrb.2017.01.024 28129563

[114] Mirili C Bilici M. Inflammatory Prognostic Markers in Endometrial Carcinoma: Systemic Immune-Inflammation Index and Prognostic Nutritional Index. Med Sci Discov (2020) 7(1):351–9. doi: 10.36472/msd.v7i1.339

[115] Aoyama T Takano M Miyamoto M Yoshikawa T Kato K Sakamoto T. Pretreatment Neutrophil-to-Lymphocyte Ratio was a Predictor of Lymph Node Metastasis in Endometrial Cancer Patients. Oncology (2019) 96(5):259–67. doi: 10.1159/000497184 30893700

[116] Acikgoz AS Cakmak B Tuten A Oncul M Eskalen S Demirkiran F. Can Preoperative Neutrophil to Lymphocyte and Platelet to Lymphocyte Ratios Predict Cervical Stromal Involvement in Endometrioid Endometrial Adenocarcinoma? Eur J Gynaecol Oncol (2017) 38(1):20–4. 29767859

[117] Pergialiotis V Oikonomou M Damaskou V Kalantzis D Chrelias C Tsantes AE. Platelet to Lymphocyte and Neutrophil to Lymphocyte Ratio as Predictive Indices of Endometrial Carcinoma: Findings From a Retrospective Series of Patients and Meta-Analysis. J Gynecol Obstet Hum Reprod (2018) 47(10):511–6. doi: 10.1016/j.jogoh.2018.08.016 30153505

[118] Hoivik EA Hodneland E Dybvik JA Wagner-Larsen KS Fasmer KE Berg HF. A Radiogenomics Application for Prognostic Profiling of Endometrial Cancer. Commun Biol (2021) 4(1):1–12. doi: 10.1038/s42003-021-02894-5 34873276 PMC8648740

[119] Lambin P Leijenaar RTH Deist TM Peerlings J De Jong EEC Van Timmeren J. Radiomics: The Bridge Between Medical Imaging and Personalized Medicine. Nat Rev Clin Oncol (2017) 14(12):749–62. doi: 10.1038/nrclinonc.2017.141 28975929

[120] Yan BC Li Y Ma FH Zhang GF Feng F Sun MH. Radiologists With MRI-Based Radiomics Aids to Predict the Pelvic Lymph Node Metastasis in Endometrial Cancer: A Multicenter Study. Eur Radiol (2021) 31(1):411–22. doi: 10.1007/s00330-020-07099-8 32749583

[121] Veeraraghavan H Friedman CF DeLair DF Ninčević J Himoto Y Bruni SG. Machine Learning-Based Prediction of Microsatellite Instability and High Tumor Mutation Burden From Contrast-Enhanced Computed Tomography in Endometrial Cancers. Sci Rep (2020) 10(1):1–10. doi: 10.1038/s41598-020-72475-9 33082371 PMC7575573

[122] Xu X Li H Wang S Fang M Zhong L Fan W. Multiplanar MRI-Based Predictive Model for Preoperative Assessment of Lymph Node Metastasis in Endometrial Cancer. Front Oncol (2019) 9:1007. doi: 10.3389/fonc.2019.01007 31649877 PMC6794606

[123] Fasmer KE Hodneland E Dybvik JA Wagner-Larsen K Trovik J Salvesen Ø. Whole-Volume Tumor MRI Radiomics for Prognostic Modeling in Endometrial Cancer. J Magn Reson Imaging (2021) 53(3):928–37. doi: 10.1002/jmri.27444 PMC7894560 33200420

[124] Chen J Gu H Fan W Wang Y Chen S Chen X. MRI-Based Radiomic Model for Preoperative Risk Stratification in Stage I Endometrial Cancer. J Cancer (2021) 12(3):726. doi: 10.7150/jca.50872 33403030 PMC7778535

[125] van Dijk LV Fuller CD. Artificial Intelligence and Radiomics in Head and Neck Cancer Care: Opportunities, Mechanics, and Challenges. Am Soc Clin Oncol Educ B (2021) 41:e225–35. doi: 10.1200/EDBK_320951 PMC8218312 33929877

[126] Binder PS Mutch DG. Update on Prognostic Markers for Endometrial Cancer. Womens Health (2014) 10(3):277–88. doi: 10.2217/WHE.14.13 24956294

[127] Lee NK Cheung MK Shin JY Husain A Teng NN Berek JS. Prognostic Factors for Uterine Cancer in Reproductive-Aged Women. Obstet Gynecol (2007) 109(3):655–62. doi: 10.1097/01.AOG.0000255980.88205.15 17329517

[128] Koual M Ngo C Girault A Lécuru F Bats A-S. Endometrial Cancer in the Elderly: Does Age Influence Surgical Treatments, Outcomes, and Prognosis? Menopause (2018) 25(9):968–76. doi: 10.1097/GME.0000000000001119 29762198

[129] Lachance JA Everett EN Greer B Mandel L Swisher E Tamimi H. The Effect of Age on Clinical/Pathologic Features, Surgical Morbidity, and Outcome in Patients With Endometrial Cancer. Gynecol Oncol (2006) 101(3):470–5. doi: 10.1016/j.ygyno.2005.11.009 16413048

[130] Son J Carr C Yao M Radeva M Priyadarshini A Marquard J. Endometrial Cancer in Young Women: Prognostic Factors and Treatment Outcomes in Women Aged≤ 40 Years. Int J Gynecol Cancer (2020) 30(5):631–9. doi: 10.1136/ijgc-2019-001105 32213530

[131] Zeng XZ Lavoue V Lau S Press JZ Abitbol J Gotlieb R. Outcome of Robotic Surgery for Endometrial Cancer as a Function of Patient Age. Int J Gynecol Cancer (2015) 25(4):637–44. doi: 10.1097/IGC.0000000000000411 25723778

[132] Jolly S Vargas CE Kumar T Weiner SA Brabbins DS Chen PY. The Impact of Age on Long-Term Outcome in Patients With Endometrial Cancer Treated With Postoperative Radiation. Gynecol Oncol (2006) 103(1):87–93. doi: 10.1016/j.ygyno.2006.01.038 16545441

[133] Zaino RJ Kurman RJ Diana KL Morrow CP. Pathologic Models to Predict Outcome for Women With Endometrial Adenocarcinoma: The Importance of the Distinction Between Surgical Stage and Clinical Stage–A Gynecologic Oncology Group Study. Cancer Interdiscip Int J Am Cancer Soc (1996) 77(6):1115–21. doi: 10.1002/(SICI)1097-0142(19960315)77:6<1115::AID-CNCR17>3.0.CO;2-4 8635132

[134] Keys HM Roberts JA Brunetto VL Zaino RJ Spirtos NM Bloss JD. A Phase III Trial of Surgery With or Without Adjunctive External Pelvic Radiation Therapy in Intermediate Risk Endometrial Adenocarcinoma: A Gynecologic Oncology Group Study. Gynecol Oncol (2004) 92(3):744–51. doi: 10.1016/j.ygyno.2003.11.048 14984936

[135] Lurain JR Rice BL Rademaker AW Poggensee LE Schink JC Miller DS. Prognostic Factors Associated With Recurrence in Clinical Stage I Adenocarcinoma of the Endometrium. Obstet Gynecol (1991) 78(1):63–9. 2047070

[136] Crosbie EJ Zwahlen M Kitchener HC Egger M Renehan AG. Body Mass Index, Hormone Replacement Therapy, and Endometrial Cancer Risk: A Meta-Analysis. Cancer Epidemiol Prev Biomarkers (2010) 19(12):3119–30. doi: 10.1158/1055-9965.EPI-10-0832 21030602

[137] Lundqvist EÅ. Classification of Endometrial Cancer. In: Management of Endometrial Cancer. Switzerland: Springer (2020). p. 3–6. doi: 10.1007/978-3-319-64513-1_1

[138] Kitson SJ Crosbie EJ. Endometrial Cancer and Obesity. Obstet Gynaecol (2019) 21(4):237–45. doi: 10.1111/tog.12601

[139] Secord AA Hasselblad V Von Gruenigen VE Gehrig PA Modesitt SC Bae-Jump V. Body Mass Index and Mortality in Endometrial Cancer: A Systematic Review and Meta-Analysis. Gynecol Oncol (2016) 140(1):184–90. doi: 10.1016/j.ygyno.2015.10.020 PMC7065832 26524722

[140] Ward KK Shah NR Saenz CC McHale MT Alvarez EA Plaxe SC. Cardiovascular Disease Is the Leading Cause of Death Among Endometrial Cancer Patients. Gynecol Oncol (2012) 126(2):176–9. doi: 10.1016/j.ygyno.2012.04.013 22507532

[141] Arem H Park Y Pelser C Ballard-Barbash R Irwin ML Hollenbeck A. Prediagnosis Body Mass Index, Physical Activity, and Mortality in Endometrial Cancer Patients. J Natl Cancer Inst (2013) 105(5):342–9. doi: 10.1093/jnci/djs530 PMC3589256 23297041

[142] Slawinski CGV Barriuso J Guo H Renehan AG. Obesity and Cancer Treatment Outcomes: Interpreting the Complex Evidence. Clin Oncol (2020) 32(9):591–608. doi: 10.1016/j.clon.2020.05.004 32595101

[143] Hunter RJ Navo MA Thaker PH Bodurka DC Wolf JK Smith JA. Dosing Chemotherapy in Obese Patients: Actual Versus Assigned Body Surface Area (BSA). Cancer Treat Rev (2009) 35(1):69–78. doi: 10.1016/j.ctrv.2008.07.005 18922643

[144] Simpson AN Sutradhar R Ferguson SE Robertson D Cheng SY Li Q. Perioperative Outcomes of Women With and Without Class III Obesity Undergoing Hysterectomy for Endometrioid Endometrial Cancer: A Population-Based Study. Gynecol Oncol (2020) 158(3):681–8. doi: 10.1016/j.ygyno.2020.06.480 32571681

[145] Modesitt SC Tian C Kryscio R Thigpen JT Randall ME Gallion HH. Impact of Body Mass Index on Treatment Outcomes in Endometrial Cancer Patients Receiving Doxorubicin and Cisplatin: A Gynecologic Oncology Group Study. Gynecol Oncol (2007) 105(1):59–65. doi: 10.1016/j.ygyno.2006.10.045 17150247

[146] Bouwman F Smits A Lopes A Das N Pollard A Massuger L. The Impact of BMI on Surgical Complications and Outcomes in Endometrial Cancer Surgery—An Institutional Study and Systematic Review of the Literature. Gynecol Oncol (2015) 139(2):369–76. doi: 10.1016/j.ygyno.2015.09.020 26407479

[147] Pichon B Thureau S Delpon G Barillot I Mahé MA. Obesity and Radiation: Technical Difficulties, Toxicity and Efficacy. Cancer Radiother (2013) 17(5–6):543–8. doi: 10.1016/j.canrad.2013.06.034 23969243

[148] Kitson S Ryan N MacKintosh ML Edmondson R Duffy JMN Crosbie EJ. Interventions for Weight Reduction in Obesity to Improve Survival in Women With Endometrial Cancer. Cochrane Database Syst Rev (2018) 2:1465–858. doi: 10.1002/14651858.CD012513.pub2 PMC6491136 29388687

[149] Robbins JR Gayar OH Zaki M Mahan M Buekers T Elshaikh MA. Impact of Age-Adjusted Charlson Comorbidity Score on Outcomes for Patients With Early-Stage Endometrial Cancer. Gynecol Oncol (2013) 131(3):593–7. doi: 10.1016/j.ygyno.2013.10.007 24125752

[150] Seebacher V Hofstetter G Polterauer S Reinthaller A Grimm C Schwameis R. Does Thyroid-Stimulating Hormone Influence the Prognosis of Patients With Endometrial Cancer? A Multicentre Trial. Br J Cancer (2013) 109(1):215–8. doi: 10.1038/bjc.2013.282 PMC3708572 23764750

[151] Barr CE Njoku K Hotchkies L Ryan NAJ Wan YL Davies DA. Does Clinical and Biochemical Thyroid Dysfunction Impact on Endometrial Cancer Survival Outcomes? A Prospective Database Study. Cancers (Basel) (2021) 23(2):294–303. doi: 10.3390/cancers13215444 PMC8582452 34771605

[152] Zhang Z-H Su P-Y Hao J-H Sun Y-H. The Role of Preexisting Diabetes Mellitus on Incidence and Mortality of Endometrial Cancer: A Meta-Analysis of Prospective Cohort Studies. Int J Gynecol Cancer (2013) 23(2):294–303. doi: 10.1097/IGC.0b013e31827b8430 23287960

[153] Liao C Zhang D Mungo C Tompkins DA Zeidan AM. Is Diabetes Mellitus Associated With Increased Incidence and Disease-Specific Mortality in Endometrial Cancer? A Systematic Review and Meta-Analysis of Cohort Studies. Gynecol Oncol (2014) 135(1):163–71. doi: 10.1016/j.ygyno.2014.07.095 PMC4404750 25072931

[154] Felix AS Weissfeld JL Stone RA Bowser R Chivukula M Edwards RP. Factors Associated With Type I and Type II Endometrial Cancer. Cancer Causes Control (2010) 21(11):1851–6. doi: 10.1007/s10552-010-9612-8 PMC2962676 20628804

[155] Park AB Darcy KM Tian C Casablanca Y Schinkel JK Enewold L. Racial Disparities in Survival Among Women With Endometrial Cancer in an Equal Access System. Gynecol Oncol (2021) 163(1):125–9. doi: 10.1016/j.ygyno.2021.07.022 PMC8562590 34325938

[156] Cote ML Ruterbusch JJ Olson SH Lu K Ali-Fehmi R. The Growing Burden of Endometrial Cancer: A Major Racial Disparity Affecting Black Women. Cancer Epidemiol Prev Biomarkers (2015) 24(9):1407–15. doi: 10.1158/1055-9965.EPI-15-0316 26290568

[157] Feinberg J Albright B Black J Lu L Passarelli R Gysler S. Ten-Year Comparison Study of Type 1 and 2 Endometrial Cancers: Risk Factors and Outcomes. Gynecol Obstet Invest (2019) 84(3):290–7. doi: 10.1159/000493132 30602164

[158] Smotkin D Nevadunsky NS Harris K Einstein MH Yu Y Goldberg GL. Histopathologic Differences Account for Racial Disparity in Uterine Cancer Survival. Gynecol Oncol (2012) 127(3):616–9. doi: 10.1016/j.ygyno.2012.08.025 PMC3828997 22940487

[159] Sherman ME Devesa SS. Analysis of Racial Differences in Incidence, Survival, and Mortality for Malignant Tumors of the Uterine Corpus. Cancer (2003) 98(1):176–86. doi: 10.1002/cncr.11484 12833470

[160] Long B Liu FW Bristow RE. Disparities in Uterine Cancer Epidemiology, Treatment, and Survival Among African Americans in the United States. Gynecol Oncol (2013) 130(3):652–9. doi: 10.1016/j.ygyno.2013.05.020 PMC4074587 23707671

[161] Althubiti MA. Mutation Frequencies in Endometrial Cancer Patients of Different Ethnicities and Tumor Grades: An Analytical Study. Saudi J Med Med Sci (2019) 7(1):16. doi: 10.4103/sjmms.sjmms_154_18 30787852 PMC6381847

[162] Maxwell GL Risinger JI Hayes KA Alvarez AA Dodge RK Barrett JC. Racial Disparity in the Frequency of PTEN Mutations, But Not Microsatellite Instability, in Advanced Endometrial Cancers. Clin Cancer Res (2000) 6(8):2999–3005. 10955777

[163] Clifford SL Kaminetsky CP Cirisano FD Dodge R Soper JT Clarke-Pearson DL. Racial Disparity in Overexpression of the P53 Tumor Suppressor Gene in Stage I Endometrial Cancer. Am J Obstet Gynecol (1997) 176(6):s229–32. doi: 10.1016/S0002-9378(97)70380-6 9215213

[164] Madison T Schottenfeld D James SA Schwartz AG Gruber SB. Endometrial Cancer: Socioeconomic Status and Racial/Ethnic Differences in Stage at Diagnosis, Treatment, and Survival. Am J Public Health (2004) 94(12):2104–11. doi: 10.2105/AJPH.94.12.2104 PMC1448599 15569961

[165] Bain RP Greenberg RS Chung KC. Racial Differences in Survival of Women With Endometrlal Cancer. Am J Obstet Gynecol (1987) 157(4):914–23. doi: 10.1016/S0002-9378(87)80089-3 3674166

[166] Luo LY Aviki EM Lee A Kollmeier MA Abu-Rustum NR Tsai CJ. Socioeconomic Inequality and Omission of Adjuvant Radiation Therapy in High-Risk, Early-Stage Endometrial Cancer. Gynecol Oncol (2021) 161(2):463–9. doi: 10.1016/j.ygyno.2021.01.041 PMC8084986 33597092

[167] Esnaola NF Hall BL Hosokawa PW Ayanian JZ Henderson WG Khuri SF. Race and Surgical Outcomes: It Is Not All Black and White. Ann Surg (2008) 248(4):647–55. doi: 10.1097/SLA.0b013e31818a159a 18936578

[168] Nicholas Z Hu N Ying J Soisson P Dodson M Gaffney DK. Impact of Comorbid Conditions on Survival in Endometrial Cancer. Am J Clin Oncol (2014) 37(2):131–4. doi: 10.1097/COC.0b013e318277d5f4 23241506

[169] Tarney CM Tian C Wang G Dubil EA Bateman NW Chan JK. Impact of Age at Diagnosis on Racial Disparities in Endometrial Cancer Patients. Gynecol Oncol (2018) 149(1):12–21. doi: 10.1016/j.ygyno.2017.07.145 28800945 PMC6863162

[170] Bedir A Abera SF Vordermark D Medenwald D. Socioeconomic Disparities in Endometrial Cancer Survival in Germany: A Survival Analysis Using Population-Based Cancer Registry Data. J Cancer Res Clin Oncol (2022) 1–9. doi: 10.1007/s00432-021-03908-9 35064816 PMC9015991

[171] Svanvik T Marcickiewicz J Sundfeldt K Holmberg E Strömberg U. Sociodemographic Disparities in Stage-Specific Incidences of Endometrial Cancer: A Registry-Based Study in West Sweden, 1995–2016. Acta Oncol (Madr) (2019) 58(6):845–51. doi: 10.1080/0284186X.2019.1581947 30849264

[172] Fader AN Habermann EB Hanson KT Lin JF Grendys EC Dowdy SC. Disparities in Treatment and Survival for Women With Endometrial Cancer: A Contemporary National Cancer Database Registry Analysis. Gynecol Oncol (2016) 143(1):98–104. doi: 10.1016/j.ygyno.2016.07.107 27470998

[173] Cheung MR. African American Race and Low Income Neighborhoods Decrease Cause Specific Survival of Endometrial Cancer: A SEER Analysis. Asian Pac J Cancer Prev (2013) 14(4):2567–70. doi: 10.7314/APJCP.2013.14.4.2567 23725176

[174] Gildea C Nordin A Hirschowitz L Poole J. Thirty-Day Postoperative Mortality for Endometrial Carcinoma in England: A Population-Based Study. BJOG (2016) 123(11):1853–61. doi: 10.1111/1471-0528.13917 26924123

[175] Donkers H Bekkers R Massuger L Galaal K. Socioeconomic Deprivation and Survival in Endometrial Cancer: The Effect of BMI. Gynecol Oncol (2020) 156(1):178–84. doi: 10.1016/j.ygyno.2019.10.030 31759773

[176] Njoku K Barr CE Hotchkies L Quille N Louise Wan Y Crosbie EJ. Impact of Socioeconomic Deprivation on Endometrial Cancer Survival in the North West of England: A Prospective Database Analysis. BJOG (2020) 128(7):1215–24. doi: 10.1111/1471-0528.16618 PMC8248174 33289967

